# Structural underpinnings and long-term effects of resilience in Parkinson’s disease

**DOI:** 10.1038/s41531-024-00699-x

**Published:** 2024-05-02

**Authors:** Verena Dzialas, Merle C. Hoenig, Stéphane Prange, Gérard N. Bischof, the Parkinson’s Progression Marker Initiative, Alexander Drzezga, Thilo van Eimeren

**Affiliations:** 1 University of Cologne, Faculty of Medicine and University Hospital Cologne, Department of Nuclear Medicine, 50937 Cologne, Germany; 2 University of Cologne, Faculty of Mathematics and Natural Sciences, 50923 Cologne, Germany; 3 Molecular Organization of the Brain, Institute for Neuroscience and Medicine II, Research Center Juelich, 52428 Juelich, Germany; 4 Université de Lyon, Institut des Sciences Cognitives Marc Jeannerod, CNRS, UMR, 5229 Bron, France; 5 German Center for Neurodegenerative Diseases, 53127 Bonn, Germany; 6 University of Cologne, Faculty of Medicine and University Hospital Cologne, Department of Neurology, 50937 Cologne, Germany

**Keywords:** Parkinson's disease, Motor control

## Abstract

Resilience in neuroscience generally refers to an individual’s capacity to counteract the adverse effects of a neuropathological condition. While resilience mechanisms in Alzheimer’s disease are well-investigated, knowledge regarding its quantification, neurobiological underpinnings, network adaptations, and long-term effects in Parkinson’s disease is limited. Our study involved 151 Parkinson’s patients from the Parkinson’s Progression Marker Initiative Database with available Magnetic Resonance Imaging, Dopamine Transporter Single-Photon Emission Computed Tomography scans, and clinical information. We used an improved prediction model linking neuropathology to symptom severity to estimate individual resilience levels. Higher resilience levels were associated with a more active lifestyle, increased grey matter volume in motor-associated regions, a distinct structural connectivity network and maintenance of relative motor functioning for up to a decade. Overall, the results indicate that relative maintenance of motor function in Parkinson’s patients may be associated with greater neuronal substrate, allowing higher tolerance against neurodegenerative processes through dynamic network restructuring.

## Introduction

Bradykinesia is the cardinal symptom of Parkinson’s disease and, therefore, essential for diagnosing this complex movement disorder^1^. Notably, the clinical stage is preceded by a long prodromal phase, as at least 50% of the nigrostriatal dopaminergic neurons have already degenerated at diagnosis^2,3^. Yet, the impact of dopaminergic loss on clinical expression varies across individuals. The observed disparity between the clinical expression and the extent of pathophysiological burden has fuelled concepts on resilience. Resilience refers to the individual’s ability to counteract disease-related detrimental alterations to a certain degree^4^.

Given that the concept of resilience has just emerged in the Parkinson’s disease research field, we recently aimed to harmonize terminologies and methods to study resilience mechanisms, in particular motor reserve, in this movement disorder^5^. The conceptualization of the construct of motor reserve has been aligned with the well-established cognitive reserve framework of the Alzheimer’s Association working group^4^. Briefly, resilience encompasses the concepts of brain, cognitive, and motor reserve. Brain reserve relies on the neuronal substrate, such as grey matter volume or the number of synapses. Cognitive and motor reserve, in contrast, are based on the more efficient use or restructuring of distinct functional and structural networks that permit relative maintenance of either cognitive or motor function. These active adaptations of brain networks have been linked to lifestyle^6^, genetic^7^, and other premorbid factors^8,9^. Studies utilizing dopamine transporter single photon emission computed tomography (SPECT)^10^ or longitudinal cognitive assessments^11^, for example, reported that higher education or premorbid physical activity was linked to greater tolerance against dopamine transporter loss and the risk of developing dementia in Parkinson’s disease.

However, using a single value may not be sufficient to capture the complex nature of the build-up and extent of individual resilience levels. Therefore, residuals of a linear regression have more recently been used to study resilience in Parkinson’s disease. This, so-called, residual approach was originally introduced to study cognitive reserve in Alzheimer’s disease^12^. It defines resilience as the variance in a clinical outcome variable of a regression model that is not explained by neuropathological burden and other explanatory variables, such as demographic and genetic factors. Importantly, a linear relationship between neuropathological burden and clinical symptom severity is crucial for the proper application of the residual approach. In Parkinson’s disease, the gradual loss of dopamine transporter signal has consistently been related to a predominantly linear increase in motor disabilities in early disease stages, although nonlinear effects cannot be ruled out completely. These motor disabilities can be quantified by the Unified-Parkinson’s-Disease-Rating-Scale motor-score (UPDRS-III score)^13–15^. Deviations from this linear relationship may thus provide information on the underlying individual resilience capacity. Lower observed than predicted motor disabilities (i.e., negative deviations from the regression model) thereby represent higher resilience levels, while positive deviations are associated with lower resilience levels. Using the residual approach, recent studies identified functional and white matter structural networks associated with motor reserve^16,17^. Particularly, these networks were associated with a slower longitudinal dose increase in dopamine replacement therapy over two to three years. Due to the short follow-up period, but long duration of the disease, the question of long-lasting resilience effects on quality of life remains, however, open. Nevertheless, these studies provided initial indications of the applicability of the residual approach to study resilience mechanisms in Parkinson’s disease.

Notably, since the residual approach relies on the meaningfulness of errors in the model, determining the optimal model fit by maximizing the explainable degree of variance is necessary to obtain a reliable measure of resilience^18^. Therefore, a systematic investigation of the relationship between regional dopamine transporter signal and the items of the UPDRS-III score is highly relevant. Possible influencing factors are symptom category (i.e., tremor, rigour, and akinesia), side of symptom and pathology onset, and region-specific contributions of the dopamine transporter signal loss. While some studies investigated isolated aspects of this using the former UPDRS-III score^2,14,15,19^, a holistic and systematic assessment of the updated Movement Disorder Society (MDS)-UPDRS-III score is currently lacking.

Moreover, brain networks involved in resilience mechanisms are likely influenced by a multitude of factors like genetic and environmental circumstances. However, the identified networks can only represent the mechanistic pathways that the imaging technique is capable of investigating. Hence, resilience structures based on white matter or functional networks only provide broad insights into the underlying anatomical and functional connections given the spatial and temporal resolution of current Magnetic Resonance Imaging (MRI) techniques^20,21^. Structural covariance networks may overcome these limitations by reflecting not only anatomical connections and functional interactions, but also by accounting for developmental dependencies and genetic influences^22^. Thus, structural covariance networks can reflect changes across the lifespan, like in healthy and unhealthy aging^23^ and changes induced by distinct lifestyle factors^22^. Particularly, graph theoretical considerations enable investigators to visualize and translate complex covariance patterns into graphs (networks) and meaningful biological parameters such as path length. Moreover, the parameters can be computed at distinct levels, ranging from individual brain regions and sub-networks of interest to the entirety of the brain network, facilitating comprehensive group comparisons across various scales^22,24,25^. Therefore, the combination of structural covariance networks and graph theoretical analysis offers the opportunity to study both deleterious disease-related network changes and beneficial resilience mechanisms^26^.

Given the current gaps and limitations of the residual approach and the still limited knowledge on the structural underpinnings of resilience in Parkinson’s disease, the aims of this study were three-fold:

First, we aimed to identify an optimal prediction model between the MDS-UPDRS-III score and regional dopamine transporter signal to derive the most suitable residuals as a measure of resilience. Variables for this model were identified using a multiple correlational approach in a cohort of de novo Parkinson’s disease patients. Second, resilience-related structural differences in grey matter volume and regional properties of structural covariance networks were investigated. To achieve this, structural MRI scans were employed in voxel-wise grey matter volume comparisons and graph theoretical considerations of the structural covariance networks. Third, we investigated if the level of resilience directly interacts with the rate of disease progression by using a linear mixed model of the most extended follow-up period (i.e., 7 years) to date. We hypothesized that higher resilience is associated with increased grey matter volume in motor-associated regions and differences in regional properties of structural covariance networks. These differences could be due to a more active lifestyle, so we assumed that patients with high resilience report higher daily physical activity levels than patients with low resilience. Moreover, we expected that motor progression would differ significantly between patients with high and low resilience levels.

## Results

### Patient characteristics

151 patients were included, out of which *n* = 50 were categorized as high and *n* = 45 as low resilience patients based on the residual split at ±0.5 standard deviations (SD). The groups did not differ regarding age, sex, years of education, Montreal Cognitive Assessment (MoCA) test, total intracranial volume and the dopamine transporter signal of the putamen and caudate nucleus. At least in part by design, the groups differed in MDS-UPDRS-III total score. For more information, see detailed group demographics and statistics in Table 1.

**Table 1:** Demographic and clinical characteristics of patients with Parkinson’s disease

	Entire Parkinson’s disease cohort			Group comparison -high vs. low resilience-		
	Characteristics		Average ± sd	High resilience average ± sd	Low resilience average ± sd	*P* value^*^
Demographics	Number		151	50	45	
	Sex (M/F)		89/62	27/23	23/22	0.94
	Age, y		58.7 ± 4.5	58.6 ± 4.0	58.9 ± 4.8	0.30
	Education, y		15.6 ± 3.0	15.4 ± 3.4	15.7 ± 2.7	0.37
	MoCA		27.3 ± 2.0	27.4 ± 2.4	27.3 ± 1.8	0.23
	TIV		1480.8 ± 149.6	1478.3 ± 138.4	1450.1 ± 159.7	0.17
	Handedness (left/ right/ mixed)		19 / 128 / 4	6 / 43 / 1	10 / 32 / 3	0.19
	More affected side (left / right / no)		65 / 85 / 1	20 / 30 / 0	22 / 22 / 1	0.36
Dopamine transporter (SBR) hemisphere	Mean	Caudate	2.00 ± 0.55	1.96 ± 0.53	1.98 ± 0.58	0.35
		Putamen	0.82 ± 0.27	0.86 ± 0.29	0.83 ± 0.31	0.16
		Striatum	1.41 ± 0.38	1.41 ± 0.40	1.40 ± 0.43	0.41
	More affected	Caudate	1.83 ± 0.53	1.78 ± 0.52	1.79 ± 0.53	0.32
		Putamen	0.67 ± 0.22	0.71 ± 0.24	0.67 ± 0.23	0.33
		Striatum	1.25 ± 0.35	1.24 ± 0.36	1.23 ± 0.36	0.39
	Less affected	Caudate	2.18 ± 0.60	2.15 ± 0.57	2.18 ± 0.67	0.41
		Putamen	0.96 ± 0.37	1.00 ± 0.38	0.98 ± 0.44	0.15
		Striatum	1.57 ± 0.45	1.57 ± 0.45	1.58 ± 0.52	0.34
MDS-UPDRS-III bodyside	Total	Both	20.1 ± 7.8	12.5 ± 3.9	28.0 ± 6.4	< 0.001
		Less affected	9.2 ± 5.6	3.9 ± 1.9	15.2 ± 5.1	< 0.001
		More affected	17.1 ± 5.3	12.2 ± 3.7	21.3 ± 4.0	<0.001
	Limb-akinetic-rigid	Both	13.0 ± 6.4	7.7 ± 3.1	19.5 ± 6.0	< 0.001
		Less affected	4.4 ± 4.2	0.9 ± 0.6	9.0 ± 4.5	< 0.001
		More affected	10.5 ± 4.0	7.6 ± 3.0	13.5 ± 3.2	<0.001
	Tremor	Both	4.1 ± 2.7	3.3 ± 2.2	4.1 ± 2.9	0.07
		Less affected	1.8 ± 1.4	1.4 ± 1.1	1.8 ± 1.7	0.14
		More affected	3.6 ± 2.4	3.1 ± 2.1	3.5 ± 2.4	0.21
	Axial		3.0 ± 1.8	1.6 ± 1.1	4.3 ± 1.6	< 0.001

*MDS-UPDRS* Movement Disorder Society—Unified-Parkinson’s-Disease-Rating-Scale, *MoCA* Montreal Cognitive Assessment test, *SBR* Specific Binding Ratio, *SD* Standard deviation, *TIV* Total Intracranial Volume.

Patients’ characteristics in the entire Parkinson’s disease cohort are summarized as mean ± standard deviation.

*Patients with low (>0.5 standard deviation) or high resilience (<–0.5 standard deviation) were identified and compared using Mann–Whitney-U for continuous and chi-square tests for categorical variables.

### Association of regional dopamine transporter signal and MDS-UPDRS-III sub-scores

**Fig. 1:**
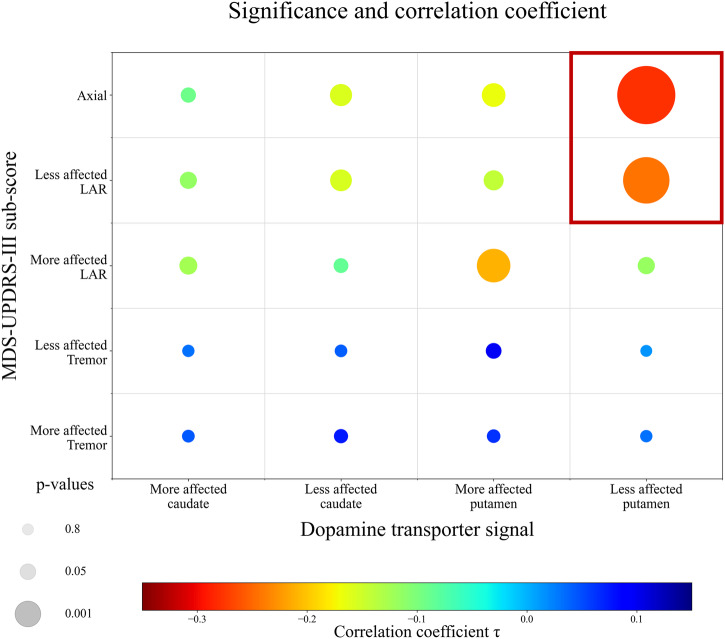
Correlation matrix visualizing *p* values and correlation coefficients by size and colour, respectively. Correlation matrix (non-parametric Kendall partial rank tau-b correlations, τ) of regional dopamine transporter signal and MDS-UPDRS-III sub-scores, with the size representing the significance level and the colour visualizing the correlation strength. A reciprocal transformation of the *p* values was performed to show the most significant correlations with the largest circles. MDS-UPDRS-III = Movement Disorder Society – Unified-Parkinson’s-Disease-Rating-Scale motor-score.

Investigating the relationship between regional dopamine transporter signal and MDS-UPDRS-III sub-scores revealed:

1. Correlations, including the putaminal dopamine transporter signal (τ = –0.19, *p* < 0.001) are more strongly associated with the MDS-UPDRS-III total score than the dopamine transporter signal of the caudate nucleus (τ = –0.11 *p* = 0.06, t = –2.35, *p* = 0.01).
2. Correlations including the axial and limb-akinetic-rigid (LAR) MDS-UPDRS-III sub-scores are more strongly associated with the dopamine transporter signal of the putamen than the tremor sub-score (t_axial_vs_tremor_ = –4.73, *p*_*axial_vs_tremor*_ < 0.001, t_LAR_vs_tremor_ = –3.74, *p*_*LAR_vs_tremor*_ < 0.001). See Supplementary Information for respective correlation coefficients.
3. Both regression models predicting either the more (F(3,147) = 8.0, *p* < 0.001, r = 0.38, R² = 0.14) or less affected axial-LAR sub-score (F(3,147) = 11.4, *p* < 0.001, r = 0.43, R² = 0.19) were significant. In both models, the contralateral putaminal dopamine transporter signal emerged as a significant predictor (ß_more_affected_ = −1.1, p_more_affected_ < 0.001, 95% CI_more_affected_ = –1.6:–0.6; ß_less_affected_ = –1.0, p_less_affected_ < 0.001, 95% CI_less_affected_ = –1.4:–0.6). Further, in the less affected model, sex showed a trend toward significance (ß_less_affected_ = –0.3, p_less_affected_ = 0.05, 95% CI_less_affected_ = 0:–0.6). Despite both models being significant, we observed a 26% better model fit for the regression using the less affected hemisphere and bodyside than for the model using the more affected hemisphere and bodyside. Therefore, modelling the less affected axial-LAR MDS-UPDRS-III sub-score was regarded as more accurate. See Supplementary Information and Supplementary Fig. 1 for detailed regression model comparison.
4. Collectively, the closest correlation between dopamine transporter signal and MDS-UPDRS-III score was found between the less affected putaminal dopamine transporter signal and contralateral axial-LAR MDS-UPDRS-III sub-score (Fig. 1). The resulting regression model, which was used to derive the residuals, was statistically significant (F(3147) = 11.4, *p* < 0.001, r = 0.43, R² = 0.19).

### Association between resilience and daily physical activity

**Fig. 2:**
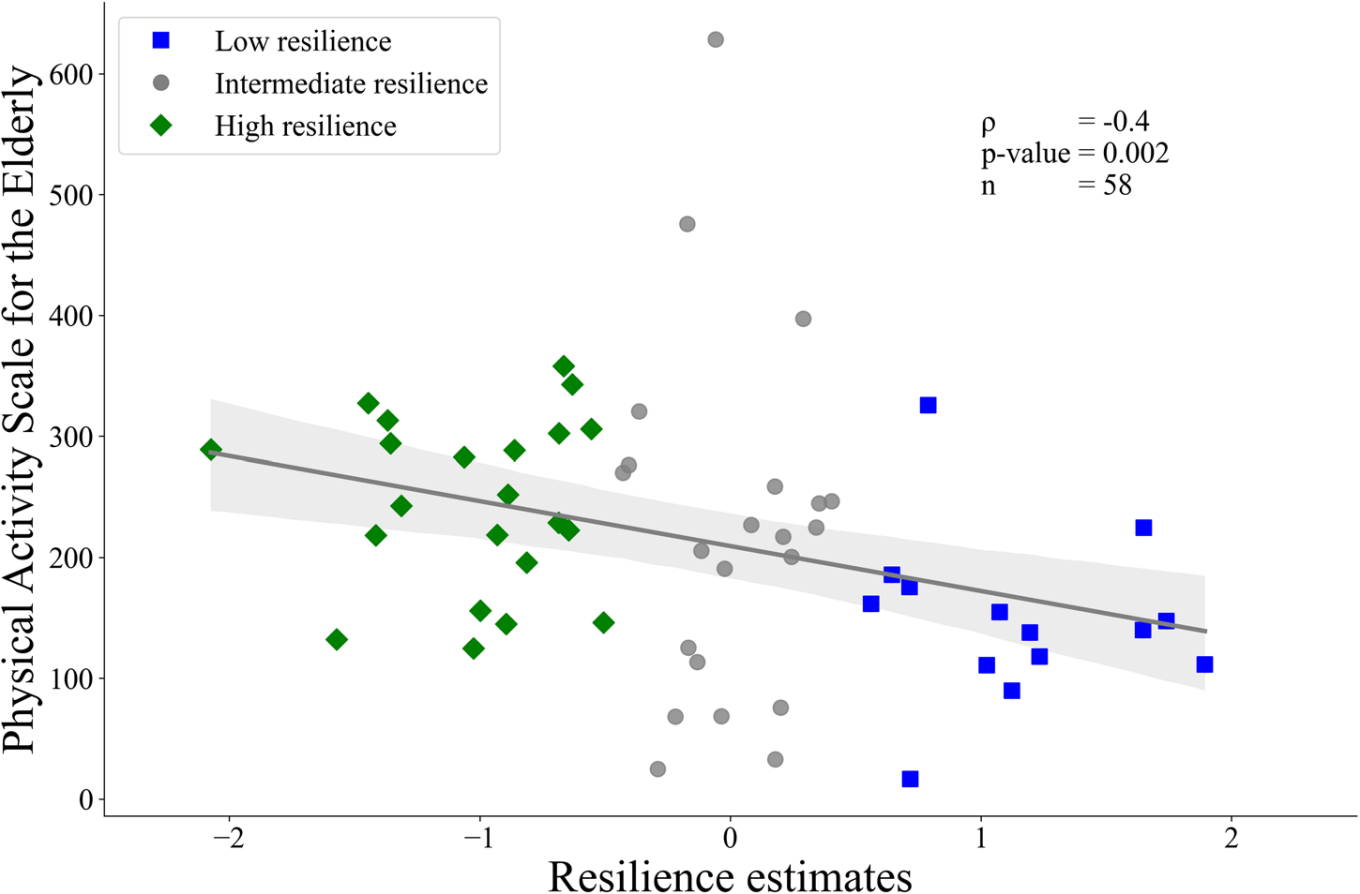
Correlation between baseline resilience values and daily physical activity. Partial Spearman correlation between baseline resilience values and the Physical Activity Scale for the Elderly at year one, corrected for age and sex is depicted. Low, intermediate, and high resilience patients are indicated by blue-filled squares, grey-filled circles, and green-filled diamonds, respectively. The plots display 95% confidence intervals as error bars, estimated using bootstrapping with 1000 iterations.

Resilience at baseline was negatively correlated with the Physical Activity Scale for the Elderly (PASE) at year one (ρ = –0.4, *p* < 0.05, Fig. 2), indicating that higher resilience is associated with greater self-reported physical activity scores. The correlations performed at later time points yielded similar trends, but only years four and seven reached the significance threshold (ρ_4_ = –0.2, ρ_7_ = –0.3, *p* < 0.05, see Supplementary Information and Supplementary Fig. 2 for correlation coefficients and significance levels).

### Morphometric differences based on resilience levels

**Fig. 3:**
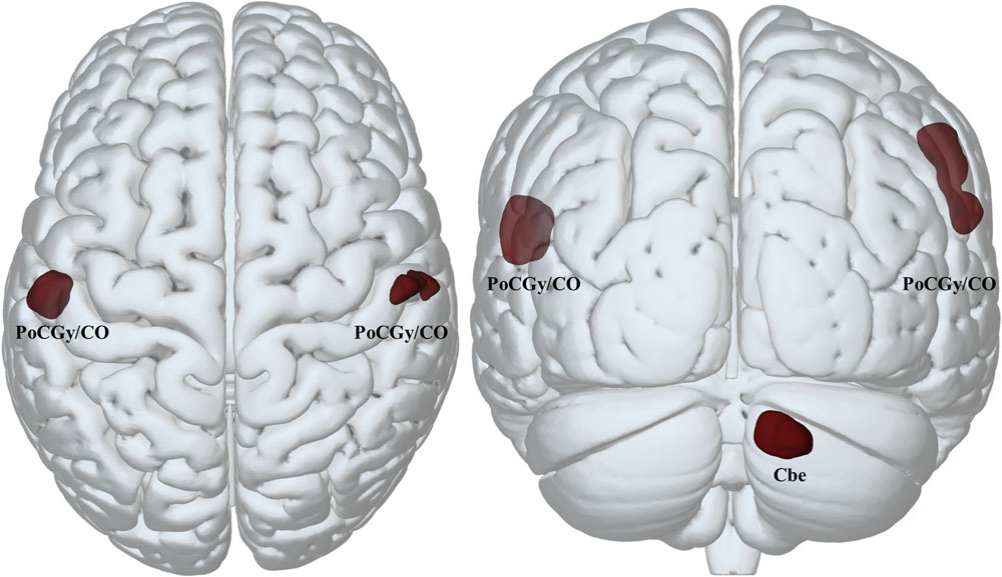
Voxel-wise grey matter volume group comparison. The high compared to the low resilience group showed greater grey matter volume in a cluster comprising the left postcentral gyrus (PoCGy) and central operculum (CO). An additional analysis, including all patients without applying the ±0.5 SD cut-off value to group the residuals, revealed the same cluster and additionally clusters in the right posterior cerebellum (Cbe) and right POCGy/CO. No significant clusters of increased grey matter volume were found with reversed contrasts. All clusters shown here were significant at cluster-level after FWE-correction (*p* *<* 0.05) with an initial *p* value set at *p* *<* 0.001.

The comparison of grey matter volume between high and low resilience patients yielded greater volume in motor-associated brain regions in the high resilience group (Fig. 3). The regions consisted of the postcentral gyrus and central operculum on the left hemisphere. The second analysis, including all patients without applying the ±0.5 SD cut-off value to group the residuals, additionally showed that the right posterior cerebellum and the right postcentral gyrus together with the central operculum were associated with higher resilience. In both analyses, no clusters of increased grey matter volume were found in the low resilience group. Considering the dominant affected bodyside as a covariate did not change the analyses results. However, the extent of the clusters varied slightly, as shown in Supplementary Fig. 3.

### Structural covariance networks of high and low resilience groups

**Fig. 4:**
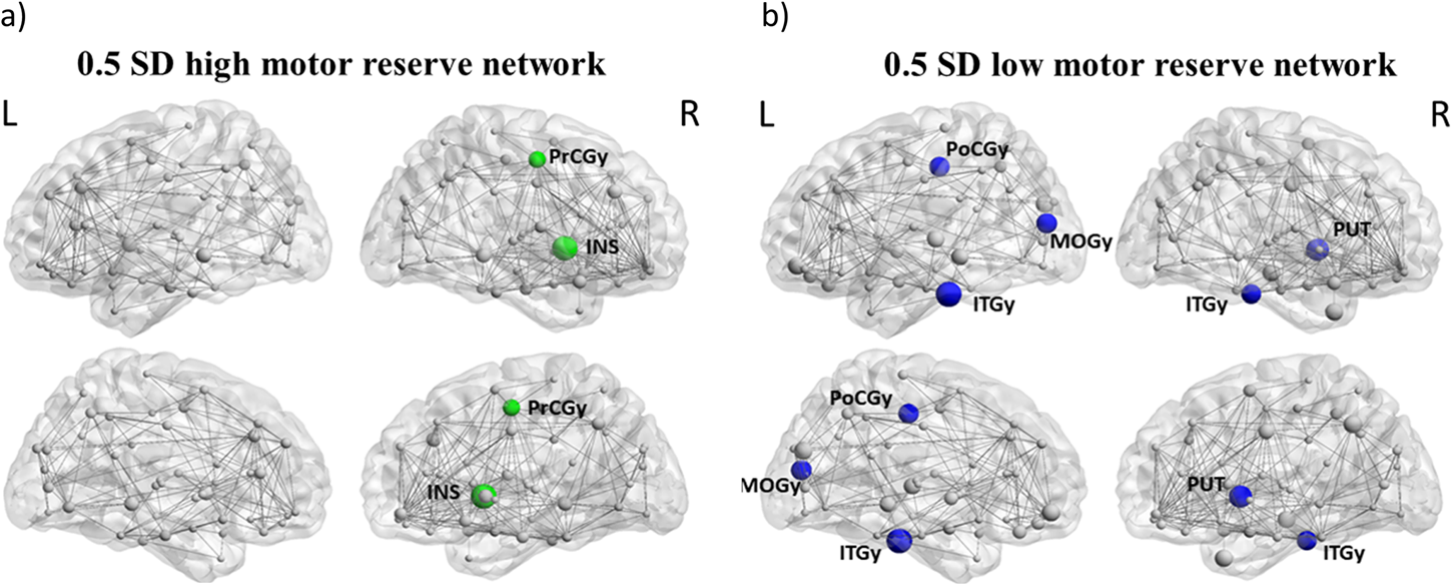
Structural covariance networks. Structural covariance networks and corresponding betweenness centrality hubs of the high (**a**—green nodes) and low resilience (**b**—blue nodes) groups, respectively. All coloured nodes show a node betweenness of >2 standard deviations of the average betweenness centrality. All hubs were verified by leave-one-out cross-validation. INS INSula, PrCGy PreCentral Gyrus, PoCGy PostCentral Gyrus, MOGy Medial Occipital Gyrus, ITGy inferior temporal gyrus, PUT PUTamen.

The regional covariance network analysis yielded robust betweenness centrality hubs in the right insula and precentral gyrus in high resilience patients (Fig. 4a). In contrast, hubs in the low resilience groups were located in the left medial occipital and postcentral gyrus, the left and right inferior temporal gyrus, and right putamen (Fig. 4b). All hubs were verified by leave-one-out cross-validation.

### Mitigating effects of resilience on disease progression

**Fig. 5:**
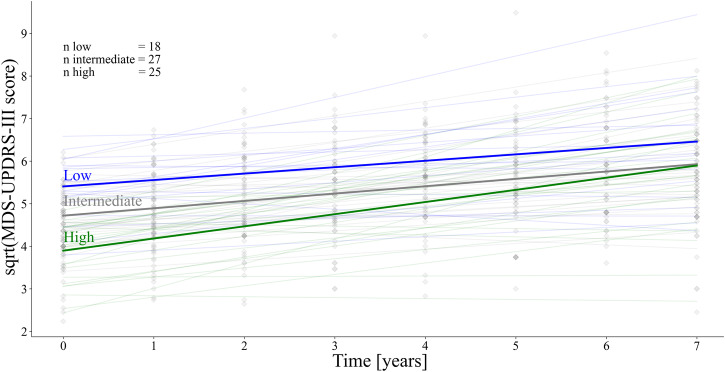
Resilience group specific decline rates and mean effects on the MDS-UPDRS-III score. Depicted are the original square root transformed MDS-UPDRS-III values in the off-medication state over time for each patient. In the background, the individual lines for each patient are shown in pale colours. In addition, the group-specific decline over time is shown (blue=low, grey=intermediate and green=high resilience). MDS-UPDRS-III = Movement Disorder Society – Unified-Parkinson’s-Disease-Rating-Scale motor-score.

The linear mixed model analyses predicted MDS-UPDRS-III off-medication (UPDRS-III-OFF) scores of high, low, and intermediate resilience patients over a seven-year follow-up period. From these models, we achieved resilience group-specific average decline rates and mean effects of the resilience groups on the UPDRS-III-OFF score. The results for predictions regarding either the total UPDRS-III-OFF score, or the more or less affected axial and LAR UPDRS-III-OFF sub-scores, showed comparable results. Consequently, high resilience patients could, on average, benefit for more than a decade (11.9 years, CI 1.4:22.5, Fig. 5) from the initial lower motor disabilities (UPDRS-III-OFF score at year 0). This extrapolation assumed that the steeper decline rates of high resilience patients remained constant even after the seven-year follow-up interval.

When comparing the decline rates in the more and less affected axial-LAR UPDRS-III-OFF sub-score models, higher decline rates could be observed in the less (ß_slope_high_resilience_ = 0.164, CI = 0.08:0.25, *p* < 0.001) compared to the more affected side model (ß_slope_high_resilience_ = 0.103, CI = 0.03:0.18, *p* < 0.05). This might point towards flooring effects that potentially dampen the steeper decline rates of high resilience patients over time. Therefore, the time interval might even be prolonged till they catch up to the impairment level of the low resilience patients. See Supplementary Table 1 for details on fixed and random unstandardized ß-coefficients. The results of the analyses did not change when adding the levodopa equivalent daily dose (LEDD) at each follow-up time point and MoCA baseline scores as covariates (for details, see Supplementary Information and Supplementary Table 2).

Analyses regarding the time until the onset of levodopa-induced dyskinesias showed a trend of the high resilience groups presenting a slight prolongation of symptom onset. However, the results did not reach statistical significance (α = 0.05 for detailed statistical analyses see Supplementary Information and Supplementary Fig. 4).

### Resilience and longitudinal dopamine signal loss

We investigated the dopamine transporter signal decline over time to exclude differences in the trajectories of dopaminergic neuron degeneration as the underlying reason for the observed longitudinal effects of resilience. Again, the mean, contra-, and ipsilateral putaminal dopamine transporter signal decline was modelled separately to consider possible laterality-related differences. The mean effects of the resilience group on the dopamine transporter signal as well as the time by resilience group interaction terms (slope), were not significant (*p* > 0.05 for details, see Supplementary Information and Supplementary Table 3). These results indicate that the differences in symptom severity and decline rates are not based on diverging dopamine transporter availability in the putamen. The only variables significantly contributing to the model were time and quadratic time in years (*p* < 0.001). Further, a significant (*p* < 0.05) covariance between the two random effects, subject (intercept) and time (slope), was observed. This covariance indicates faster dopamine transporter signal decline in patients with higher initial dopamine transporter levels independent of the resilience group.

## Discussion

Research on resilience in Parkinson’s disease is still at an early stage. Therefore, methodological aspects for the quantification of resilience, as well as its neurobiological underpinnings and moderating effects in the longitudinal disease trajectory, are highly relevant. Using the systematically-derived residuals as resilience estimates, this study identified key brain regions for motor information processing involved in the mitigation of detrimental disease effects. Higher resilience (i.e., negative residual value) was associated with hubs in the right precentral gyrus and insula and increased grey matter volume in the bilateral postcentral gyrus and right cerebellum. Importantly, even though higher resilience levels were associated with increased functionality at baseline, longitudinal analysis revealed steeper decline rates in this group. Nonetheless, extrapolations indicated that high resilience patients uphold relatively high motor function for up to a decade before deteriorating to lower resilience performance levels. The strong correlation between self-reported physical activity and resilience levels further supports the beneficial effects of higher resilience on motor functions. Notably, compared to other studies, we closely examined the relationship between the variables from which the resilience estimates were derived. The implications of determining the optimal model for quantifying resilience estimates will be discussed in the next section.

To determine resilience by means of the residual approach, a non-invasive neuropathological measure (i.e., dopamine transporter SPECT) is required to predict the clinically overt motor disabilities (i.e., MDS-UPDRS-III score) as accurately as possible. Our systematic assessment found the strongest association between the putaminal dopamine transporter signal anatomically contralateral to the less affected axial-LAR MDS-UPDRS-III sub-score.

Previous studies using the residual approach to determine individual resilience levels focused on the UPDRS-III total score and putaminal dopamine transporter signal and neglected symptom laterality and symptom categories^16,17,27,28^. However, there is an increasing body of evidence reporting that tremor is not associated with nigrostriatal degeneration^29^, while the putaminal dopamine transporter signal is most strongly related to certain sub-scores of the UPDRS-III score^14,15,19^. In addition, patients with left-sided symptom onset showed greater symptom progression over time^30^, suggesting that laterality should be considered when estimating resilience.

In line with previous studies investigating the association between the dopamine transporter signal and UPDRS-III items, we found significant differences in the correlation strength regarding striatal sub-regions, MDS-UPDRS-III items and symptom laterality. Still, one might have expected a closer association between the more affected bodyside and contralateral hemisphere. However, flooring effects can limit the variance in dopamine transporter signals in correlation and regression models reducing both the correlation strength and power to determine inter-individual differences in resilience levels. As demonstrated in previous studies, these flooring effects influence the more affected side earlier and more intensely^31^, while the general relationship between radiotracers and dopaminergic cell loss dropped when neurodegeneration exceeded 50%^32^. Therefore, using the symptom severity of the less affected bodyside and anatomically contralateral dopamine transporter signal may potentially provide a more precise measure of resilience. Nonetheless, early preclinical stages may exhibit reversed effects, yet accurately assessing this possibility is challenging due to the limited sensitivity of the MDS-UPDRS-III score in detecting subtle motor dysfunction. As a proof of concept for our model, residuals showed a strong positive association with self-reported daily physical activity levels. Physical activity, therefore, may provide an essential factor in enhancing neuroprotective and -plasticity mechanisms, enabling the relative preservation of motor function despite striatal dopamine loss^33,34^. Together with the resilience-dependent differences in grey matter volume and structural connectivity networks, this points towards an interplay between more passive (brain reserve) and active (motor reserve) mechanisms, leading to combined resilience effects, which will be further discussed below.

Brain reserve and motor reserve are closely intertwined domains of resilience. While brain reserve provides the more or less robust basis for structural and functional connectivity, motor reserve uses this biological basis to adapt brain networks to task demands in face of disease-related conditions^5^. This close interconnection makes it necessary to attempt to decipher the specific contribution of each domain to the clinical phenotype, while also considering their interdependence. In our study, we observed that patients with higher resilience were characterized by greater grey matter volume in motor-associated brain regions (i.e., brain reserve) but also differences in betweenness centrality relating to the efficiency of information processing in (sub)cortical networks (i.e., motor reserve).

In particular, patients with higher resilience levels showed greater grey matter volume in bilateral postcentral gyri and the right cerebellum. The increased grey matter volume in these motor-associated regions likely provides greater tolerance towards impending neurodegeneration until symptom onset and the breakdown of networks involving these brain regions. In line with this, resilience estimates (residual approach) and brain reserve estimates (deformation-based morphometry) were recently shown to correlate positively with local striatal volumes^35^ and negatively with clinical measures of disease progression^35,36^. However, the results of these studies were restricted to subcortical regions, which are known to undergo severe brain atrophic processes in early stages of Parkinson’s disease^37^. Here, we used a whole-brain voxel-wise method to identify more global effects of brain reserve in cortical and cerebellar regions, which may not have yet been affected by the neurodegenerative process. Thereby, greater grey matter volume in these regions may support the actual preservation of neuronal networks.

The hub analysis of our structural covariance network analysis yielded the precentral gyrus and insula as hubs in the high resilience group. These hubs were based on betweenness centrality, a measure of the importance of a brain region for information transmission and distribution inside a network. Therefore, the hubs resemble connectors of different sub-networks integrating high amounts of information. The identified precentral gyrus is an essential part of the human motor system for movement control and decision-making, while the role of the insula in motor control and Parkinson’s disease has just recently gained more attention. Research efforts revealed insular activation to be crucial in body awareness and perception of time for complex movements^38^. Further, the insula is associated with aspects of motivation, which have recently been argued to carry an important role in voluntary movement and akinesia in Parkinson’s disease^39^. While the discussed network hubs of the high resilience group are more confined to motor regions, the low resilience group showed a more widespread pattern of significant hubs, including the left postcentral-, middle occipital and inferior temporal gyrus as well as the right putamen and inferior temporal gyrus.

Supporting the role of the insula and cerebellum in resilience mechanisms in Parkinson’s disease, a study recently identified these regions in a functional motor reserve network^16^. Additionally, the importance of the cerebellum in the maintenance of motor functioning is underpinned by its role in a resilience-related white matter network^17^. Higher connectivity in the identified networks was associated with slower disease progression. Together with our observation that the insular and precentral gyrus are the only hubs in the high resilience network, this points toward a more segregated motor network in patients with higher resilience. Segregation describes highly connected sub-networks for localized task performance, while integration refers to the cooperation between sub-networks. Given cost-efficiency, not all brain regions are equally interconnected, leading to clusters of highly connected (segregated) sub-networks which are only interconnected (integrated) by a few links^40^. Importantly, network segregation has been shown to be crucial for the execution of motor tasks, while higher cognitive functions seem to be associated with a more integrated topology^41^. Indeed, it was shown that training of specific movements leads to higher segregation of brain regions involved in motoric functioning and visual perception^42^. Although this indicates that differences in motor network segregation might be a potential explanation for performance differences of high and low resilience patients, higher segregation may also pose a liability. The benefits of independent and automatized motor task performance in early disease stages come with the cost of only a few brain regions as connectors, which makes them more vulnerable.

Overall, the results of this study may provide insights into the importance of network segregation in the relative maintenance of motor function. This, however, does not exclude the possibility that higher integration also serves as motor reserve mechanism, as recently shown^8^. To distinguish disease-induced changes from those related to reserve, a longitudinal assessment of changes in network segregation and integration is required. Such analyses could also examine the specific effects of genetic determinants of brain anatomy (i.e., brain reserve) and lifestyle factors on network stability and plasticity (i.e., motor reserve). Investigating genetic and lifelong influences on resilience could provide valuable insights into the heterogeneity of the disease pattern and thus support early diagnosis and prognosis.

Given the heterogeneity across Parkinson’s disease patients, accurate prognosis regarding the patient-specific disease course remains difficult^43^. The disease itself is influenced by a multitude of factors, such as genetics, demographics and lifestyle, which to varying degrees contribute to the build-up of the individual resilience capacity. Therefore, it seems crucial to consider potential effects of resilience on the timing of clinical diagnosis and clinical progression. Yet, in Parkinson’s disease, only few studies are available investigating the longitudinal effect of resilience mechanisms. Our longitudinal analysis of seven-year follow-up data showed that patients with higher resilience had less overall motor disabilities but steeper decline rates. However, extrapolations, assuming constant decline rates, indicate that it would take up to a decade for high resilience patients to be on par with motor performance levels of low resilience patients.

In contrast to our results, higher resilience was recently linked to slower progression in clinical scores^36^, lower risk of developing levodopa-induced dyskinesia, freezing of gait^27^ or dementia^28^ and slower dose increase in levodopa therapy^16,17^. However, another study could not find any resilience-related differences in motor performance decline rates, but similar to our study, it associated higher resilience with lower motor disabilities at baseline^44^. Differences in outcome variables, surrogate measures for resilience, and varying follow-up periods might cause these conflicting results regarding the effect of resilience on disease progression. Especially the follow-up period seems to influence the results, suggesting less beneficial long-term effects of resilience given longer follow-up periods. While short-term studies associated slower decline rates with higher resilience, our long-term study showed sustained beneficial effects that may wane after several years.

Our study differs from previous ones in terms of methodological aspects, which might explain the deviations regarding long-term effects of resilience. First, our cohort had a restricted age range and younger patients, reducing the influence of age-related network alterations and differences in dopamine transporter availability. Second, we addressed laterality-induced flooring effects in the dopamine transporter signal and ceiling effects in clinical symptom assessment. We also excluded items from the MDS-UPDRS-III score unrelated to the severity of dopaminergic deficit, increasing the validity of the residuals. Furthermore, our study investigated long-term effects of resilience over a seven-year follow-up interval, unlike previous studies, which only followed up for two to three years. Finally, we used a direct measure of disease severity (MDS-UPDRS-III score) instead of indirect measures like the LEDD dose, which might not provide an objective measure of disease progression^45^.

Noteworthy, no study to date has been able to show that patients with higher resilience in Parkinson’s, like in Alzheimer’s disease^46^, can tolerate more pathology till symptom onset. While resilience in Parkinson’s disease primarily relies on differences in clinical symptom severity, Alzheimer’s patients differ regarding the pathological load^47^. This difference might be related to differences in the pathological measure. In Alzheimer’s disease, the accumulation of harmful proteins (amyloid and tau) is used, while in Parkinson’s disease, neuronal loss serves as the neuropathological measure.

Despite our systematic investigation of the correlation between dopamine transporter signal and MDS-UPDRS-III score, several limitations must be considered. Namely, the residuals are still based on the error in the model^18,48^ and are highly correlated with the dependent variable^18^. However, our systematic model selection and restriction to young, de novo patients maximized the explainable variance in the residual approach and minimized noise caused by flooring effects. Further, we validated our resilience estimates by correlation analysis with an independent measure of daily physical activity. However, the correlation between dopamine transporter availability and MDS-UPDRS-III score remained at the lower end of the expected spectrum. The rather moderate correlation strength is likely linked to the study design. First, the multi-centre data acquisition introduces random variation. Second, the early disease state of the studied cohort limits the variability in dopamine transporter availability and, consequently, the strength of the correlation. Further, we cannot rule out that our age restriction or other factors like genetics might influence the results. It will thus be interesting to assess whether comparable neuronal imprints exist in older individuals, how they evolve as the disease advances, and to further disentangle the domains (i.e., brain and motor reserve) of resilience. Moreover, in our grey matter volume analysis, we refrained from further subdividing groups based on the dominant affected side and handedness due to the limited sample size and the resulting power issues. However, investigating these effects might provide valuable insights into laterality mechanisms related to resilience. Additionally, examining the mediating role of premorbid and current lifestyle factors explaining the derived residuals and identified network structures warrants future studies. These studies may provide novel insights for the development of interventional strategies targeting these networks.

In sum, this study demonstrated the residual approach’s usefulness in identifying resilience-related structural differences and the influence of different resilience levels on disease progression. The relative maintenance of motor function in Parkinson’s disease patients is likely driven by brain reserve, potentially allowing greater tolerance against neurodegenerative processes through motor reserve-associated network restructuring. Network restructuring may, in turn, lead to a higher segregated motor network that supports more efficient motor performance for up to a decade. However, it remains to be elucidated which factors can influence the build-up and maintenance of resilience, as the residuals used as approximation of resilience represent the sum of disease and mitigating factors after disease onset. As indicated by our correlational approach, current physical activity may play an important role in the maintenance of relative motor function. Moreover, modifiable early and midlife lifestyle parameters are of high interest but also (epi)genetic, metabolic, and proteomic factors. Future investigations may further examine the interlinkage of motor and cognitive reserve networks. Understanding whether primary motor circuitries are more efficient in mitigating motor decline rather than cognition-relevant structures or vice versa will be especially relevant for patients with mild cognitive impairment or dementia.

## Methods

### Participants

**Fig. 6:**
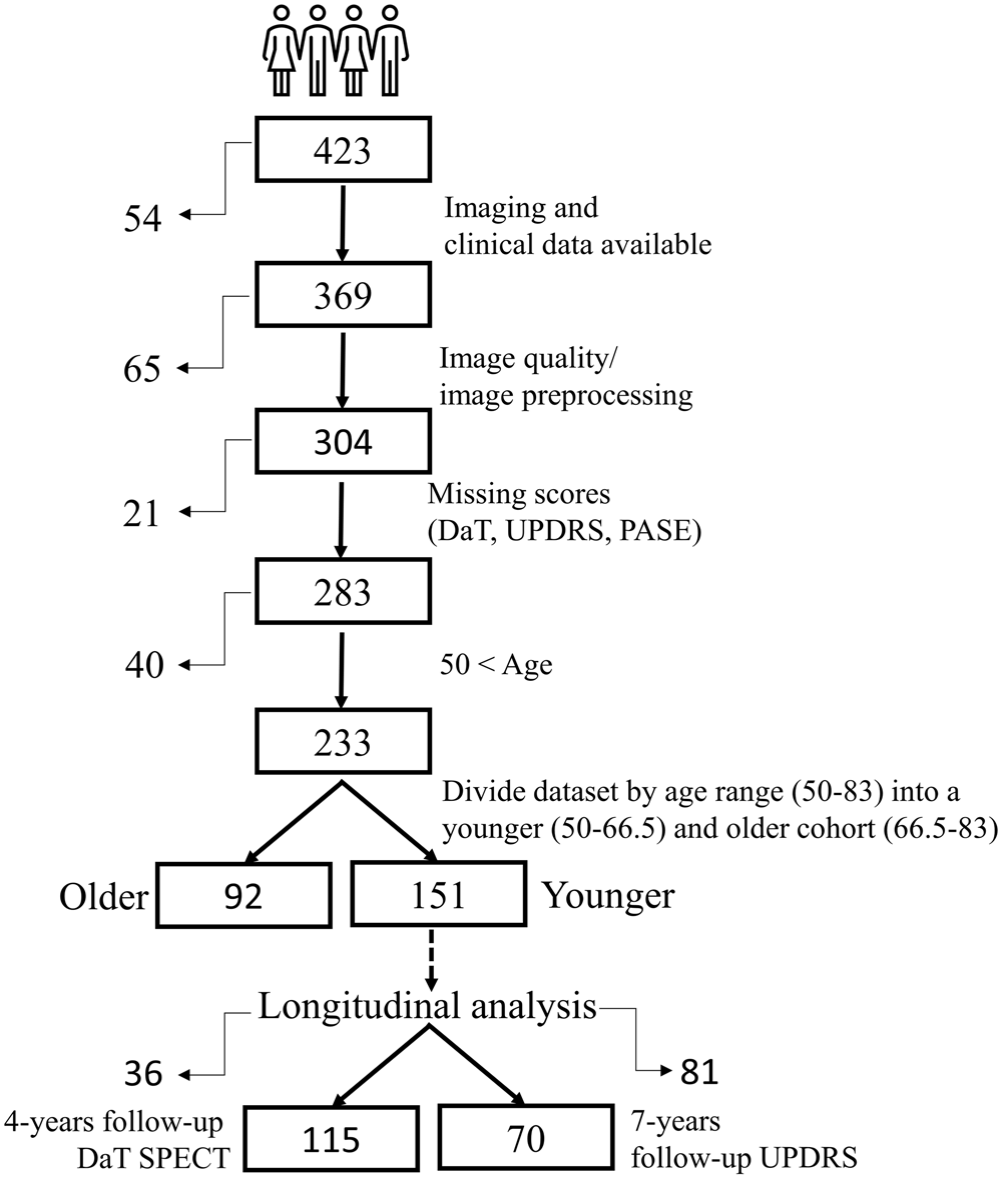
Flowchart of the application of the inclusion criteria. The filtering pipeline was started with 423 patients and included five filtering steps as described on the right side of the flowchart. On the left side the number of patients excluded in each step are stated. From the resulting 151 patients, those with a seven-year MDS-UPDRS-III score follow-up in the off-medication state (*n* = 70) and those with four-year Dopamine transporter follow-up (*n* = 115) were included in the cohorts for the longitudinal analyses. MDS-UPDRS-III Movement Disorder Society—Unified-Parkinson’s-Disease-Rating-Scale motor-score; DaT SPECT Dopamine transporter Single Photon Emission Computed Tomography.

Data used in the preparation of this article were obtained from the Parkinson’s Progression Markers Initiative (PPMI) database (www.ppmi-info.org/access-data-specimens/download-data), RRID:SCR_006431. For up-to-date information on the study, visit www.ppmi-info.org. Inclusion criteria were: (1) de novo, unmedicated Parkinson’s disease patients (at baseline) diagnosed according to clinical criteria from Postuma et al. ^1^; (2) age 50–66.5 years to avoid inclusion of familiar cases and age-related differences in the integrity of the dopaminergic system^49^; (3) available baseline dopamine transporter SPECT scan ([^123^I]β-CIT); (4) available baseline structural MRI scan; (5) at least one available PASE score. This led to the inclusion of 151 patients (see Fig. 6 for details of the filtering process and Supplementary Table 4 for the PPMI identifiers). Ethical approval and written informed consent according to the Declaration of Helsinki for all patients were obtained from the respective PPMI sites. The PPMI trial was registered under NCT01141023.

### Processing of neuroimaging data

Structural T1-weighted scans were acquired following the PPMI protocol (for detailed information, see https://www.ppmi-info.org/study-design/research-documents-and-sops), with a total scan time of seven minutes on 3 T MRI machines. The structural MRI images were segmented into grey, white, and cerebrospinal fluid compartments and normalized to the Montreal Neurological Institute space using the Computational Anatomy Toolbox in SPM12^50^. The resulting grey matter volume maps were used for subsequent analyses.

SPECT imaging using ^123^I Ioflupane ([^123^I]β-CIT) was performed according to the PPMI standardized protocol to quantify dopamine transporters in the striatum. In this study, the specific binding ratios for putamen and caudate nucleus for both hemispheres, as provided in the PPMI database, were used. Specific details about the image acquisition parameters, attenuation correction, pre-processing, and predefined variables by PPMI are available at https://www.ppmi-info.org/study-design/research-documents-and-sops.

The dopamine transporter values of the right and left caudate nucleus and putamen were extracted, using the occipital lobe as reference region. Based on the predominantly affected bodyside at onset (DOMSIDE in the PPMI datasheet), the contralateral hemisphere was labelled as more affected, while the ipsilateral hemisphere was regarded as less affected.

### Motor assessment and clinical evaluation

Due to the study design of PPMI, two MDS-UPDRS-III assessment dates were available, namely screening and baseline, which were only 1.5 months apart. To account for non-disease-related fluctuations in daily performance, we averaged the scores of both assessments. Side-specific MDS-UPDRS-III sub-scores were calculated for LAR, tremor and axial sub-scores, as done previously^15,51^. In total, five MDS-UPDRS-III sub-scores were defined, namely an axial and two for the less and more affected LAR and tremor sub-scores, respectively. For patients without a predominantly affected side at onset, the average score of both sides was computed.

### Correlations between dopamine transporter signals and MDS-UPDRS-III sub-scores

**Fig. 7:**
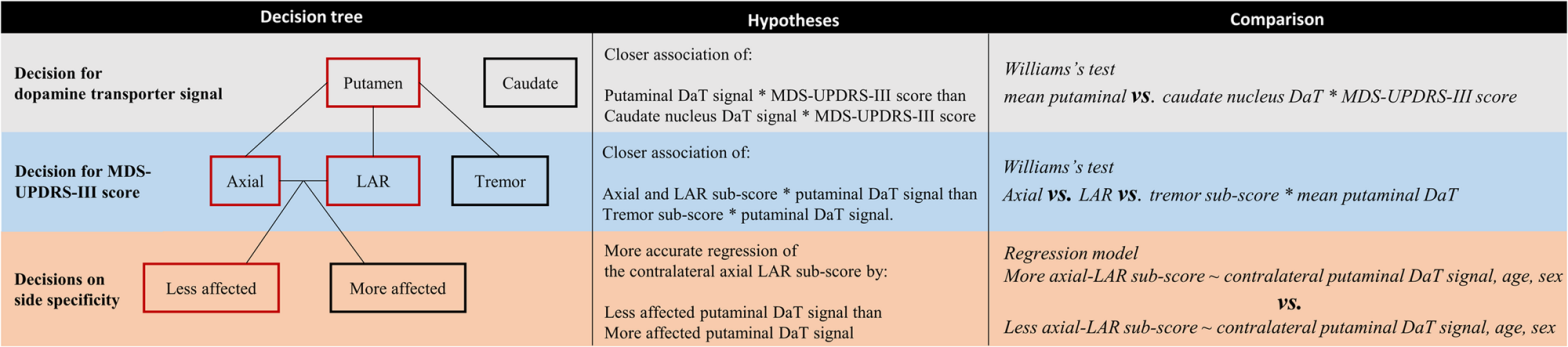
Hierarchical test cascade to identify the linear regression model with the best fit. Schematic illustration of the statistical analysis approach. Left column: The hypotheses are illustrated as decision tree; Middle column: The tested hypotheses; Right column: Performed statistical analyses to test the three hypotheses. The red path in the decision tree points to the statistically significantly stronger associations. DaT Dopamine Transporter, LAR limb-akinetic-rigid, MDS-UPDRS-III Movement Disorder Society—Unified-Parkinson’s-Disease-Rating-Scale motor-score.

We performed a hierarchical hypotheses-driven analysis to determine which model most reliably predicts motor impairments as a function of regional dopamine transporter signal. First, we performed pairwise correlations between hemispheric dopamine transporter signals in the putamen or caudate nucleus and the MDS-UPDRS-III sub-scores. We used non-parametric Kendall partial rank tau-b correlations and adjusted the analyses for age and sex. Secondly, hypotheses were formulated by visually assessing the correlation strength between the studied pairs (Fig. 1). As shown in Fig. 7, these hypotheses were then tested in a hierarchical cascade to determine the most predictive correlation. All results regarding the hypotheses testing are reported with one-tailed *p* values given the clearly defined hypotheses (for detailed information about the hypothesis testing procedure see Supplementary Information). All analyses described thus far were calculated using *RStudio version 1.3.959*^52^.

### Calculation of resilience proxy using the residual approach

Subsequently, we calculated resilience levels as the standardized linear regression residuals^16,17,27,28^, using the square root transformed combined axial and LAR MDS-UPDRS-III sub-score of the less affected bodyside as dependent variable and the contralateral putaminal dopamine transporter signal as predictor, correcting for age and sex. Negative residual values in this model indicate high resilience, while positive deviations capture individuals with low resilience. As residuals close to the regression line may mostly relate to noise, we discarded residual values around zero (*n* = 56) and grouped the remaining individuals into high (<–0.5 SD) and low resilience (>+0.5 SD from the regression line) patients. For group-specific characteristics see Table 1.

### Correlation analysis between resilience and PASE score

To determine whether higher resilience is associated with greater daily physical activity, a partial Spearman correlation analysis between the PASE scores (see https://meetinstrumentenzorg.nl/wp-content/uploads/instrumenten/PASE-handl.pdf for computation) and residual values, corrected for age and sex, was performed. Given that the baseline PASE score was only available for eight individuals, separate correlations (i.e., seven) were performed for each time point with sufficient data available (*n* > 50, for detailed information about patient per time point availability, see Supplementary Table 5). This analysis was conducted in Python version 3.8^53^.

### Voxel-wise whole brain group comparison of grey matter volume

To examine differences in grey matter volume as a function of resilience, the high resilience group was compared against the low resilience group, using voxel-wise whole-brain comparison in SPM12. The comparison was corrected for age, sex, and total intracranial volume. A whole-brain cortical mask was employed. The *p* value was set at *p* < 0.001 (uncorrected), and the voxel extent was set to k = 100. Resulting clusters significant at FWE-corrected *p* < 0.05 were then considered in the results. Reverse contrasts (low vs. high resilience) were also assessed. Arguably, the above-mentioned analyses only consider participants with strong deviations from the expected MDS-UPDRS-III score. Therefore, the analysis was repeated with a residual split at a residual value of 0 (for group characteristics, see Supplementary Table 6). Moreover, to account for a potential bias that might arise from differences in the absolute numbers of patients with left and right dominant affected sides in the high and low resilience groups, we repeated the analyses, including “dominant side” as a covariate.

### Structural brain network analysis

To compare resilience level-dependent structural network properties, we further performed structural covariance network analyses. This type of analysis can generate a graphical representation of how brain regions are structurally interconnected and estimate whole-brain or region-specific morphological measures that reflect, for example, the network’s effectiveness in information transmission. In our analysis, we investigated differences in the structural covariance networks between the two resilience groups, using the Graph Theoretical Analysis (GAT https://www.nitrc.org/projects/gat/) toolbox^24^. First, the grey matter volume-maps were corrected for age, sex, and total intracranial volume and parcellated using the Automatic Anatomical Labelling atlas resulting in 90 regions of interest. Next, association matrices (90 × 90 regions of interest) were computed, which comprised the corrected covariance values for every pair of regions of interest using leave-one-out cross-validation. For the computation of network properties, like betweenness centrality, the matrices were then thresholded at the minimum density (D_min_)_,_ resulting in non-fragmented networks of full connectivity (with D_min_ ranging from 0.1 to 0.19 for the cross-validation analyses). Betweenness centrality measures the importance of a brain region (node) for information transmission and distribution inside a network by bridging different network clusters^25^. Based on this measure, hubs were identified as nodes with a betweenness centrality greater than two standard deviations of the regular nodes within the same network^24^. Next, we compared the regional distribution of the identified hubs between the resilience groups. Given that the difference in hub distribution may be linked to network restructuring and, thus, more active forms of reserve, we refer to them as high and low motor reserve networks. By comparing the regional distribution of the identified hubs in the high and low motor-reserve networks, it is possible to detect alterations in regional network structures. Network hubs identified in over 80% of the cross-validation analyses were considered stable.

### Linear mixed modelling to assess longitudinal resilience effects

Next, we assessed if disease trajectories differ depending on baseline resilience estimates. Therefore, a linear mixed model was calculated to track longitudinal changes (seven-year follow-up) in motor performance (MDS-UPDRS-III off-medication score; i.e., UPDRS-III-OFF) in relation to the resilience group using *SPSS (IBM SPSS Statistics for Windows, Version 28.0. Armonk, NY)*. The off-medication score was used to avoid medication influences on the MDS-UPDRS-III score, especially later in the disease course. Further, only patients with a seven-year follow-up period were included in this analysis (*n* = 70; for cohort and group information, see Supplementary Table 7). The scores were averaged if multiple assessment dates were available within one year. Individuals close to the regression line within ±0.5 SD were included as an intermediate reference group. The model included the following fixed effects: time in years (continuous); (2) quadratic time in years (continuous); (3) resilience category (categorical with a high, intermediate, and low resilience category); (4) interaction term time (1) and category (3); (5) covariates of no interest (age (continuous), sex (categorical) and the putaminal dopamine transporter signal of the less affected hemisphere). The dopamine transporter signal was used to correct for potential group differences in overall dopaminergic degeneration, while the quadratic time effect accounted for non-linear decline processes. In addition, the model included two random effects (subject and time) that allowed individual intercepts and slopes. Results are reported as unstandardized beta-coefficients (ß) with the corresponding confidence intervals (CI) and *p* values.

The same modelling was repeated for the more and less affected axial-LAR UPDRS-III-OFF sub-score to investigate possible laterality-related differences in decline rates. Based on Akaike and Bayesian information criteria, the baseline dopamine transporter signal of the respective contralateral side was used for the more and less affected UPDRS-III-OFF sub-score model. We repeated the mixed model analyses, including LEDD at each follow-up time point and the baseline MoCA scores, to account for possible confounding effects of cognition and medication (for details see Supplementary Information).

As a complementary analysis, Kaplan-Meier survival curves were employed to examine resilience-related differences in the time until the onset of levodopa-induced dyskinesias. Additional analysis and data availability details can be found in Supplementary Information and Supplementary Table 8.

### Linear mixed model-based extrapolation of long-term effects

Considering the more rapid decline in cognitive function in patients with high cognitive reserve from the time of diagnosis of Alzheimer’s disease^54^, we assumed faster motor decline of high resilience patients in Parkinson’s disease as well. The time they need to catch up to the level of motor disabilities of low resilience patients can be estimated via formula 1 (1). Next, we extrapolated the time interval during which patients can benefit from higher resilience. We did this by dividing the difference between the initial motor disabilities of high and low resilience patients (UPDRS-III-OFF score_year 0_ of the low resilience group (a) and the high resilience group (b)) by the difference in decline rates (slopes of the low resilience group (c) and the high resilience group (d)).

1 $$\frac{{{Year}\,0\,{UPDRS}}_{{low}\,{motor}\,{reserve}}-{{Year}\,0\,{UPDRS}}_{{high}\,{motor}\,{reserve}}}{{{Decline}\,{rate}}_{{low}\,{motor}\,{reserve}}-{{Decline}\,{rate}}_{{high}\,{motor}\,{reserve}}}=\frac{a-b}{c-d}=\frac{e}{f}={years}\,{of}\,{benefit}$$

Confidence intervals were computed via error propagation using the Eq. (2), based on the standard errors (δ) of the unstandardized beta coefficients:

2 $${error}\,{propagation}\frac{e}{f}=\frac{e}{f}\sqrt{\left(\frac{\delta e}{e}\right)+\left(\frac{\delta f}{f}\right)}=\left(\frac{a-b}{c-d}\right)\sqrt{\left(\frac{\sqrt{{(\delta a)}^{2}+{(\delta b)}^{2}}}{(a-b)}\right)+\left(\frac{\sqrt{{(\delta c)}^{2}+{(\delta d)}^{2}}}{(c-d)}\right)}$$

### Resilience category-dependent longitudinal dopamine transporter signal decline

To exclude resilience category-dependent differences in the pace of neuropathological changes, an additional linear mixed model was performed, investigating the dopamine transporter signal decline over time. The model was set up using the same fixed and random effects, except for the baseline putaminal dopamine transporter signal. In contrast to the MDS-UPDRS-III score, dopamine transporter imaging was only assessed till year four, not seven. Therefore, in this analysis, all patients with less than four-year follow-up data were excluded (for cohort and group information, see Supplementary Table 9). Again, to account for side related differences, the model was computed three times, for the more affected, mean and less affected putamen.

All statistical tests report two-sided *p* values unless otherwise stated.

### Reporting summary

Further information on research design is available in the Nature Research Reporting Summary linked to this article.

### Supplementary information

Supplementary Information

Reporting Summary

## Data Availability

The data used for this study are publicly available via the PPMI website (https://www.ppmi-info.org/). Unique identifiers of the subjects included in this study can be found in Supplementary Table 4.
