# Supplementary material for: Structural underpinnings and long-term effects of resilience in Parkinson’s disease
Source: NPJ Parkinsons Dis. 2024 May 2;10:94. doi: 10.1038/s41531-024-00699-x (PMC11066097; doi:10.1038/s41531-024-00699-x)
Supplement: Supplementary file 1 — Supplementary Information [file 41531_2024_699_MOESM1_ESM.pdf]

Supplementary Information

Results

Association of regional dopamine transporter signal and MDS-UPDRS-III sub-scores

The hypotheses tests validated that:

1) The putaminal dopamine transporter signal (τ=-0.19, $p<0.001$) is significantly closer associated with the MDS-UPDRS-III score (t=-2.35, p=0.01) than the signal of the caudate nucleus (τ=-0.11 p=0.06).
2) The axial and limb-akinetic-rigid (LAR) MDS-UPDRS-III sub-scores are significantly closer associated with the dopamine transporter signal of the putamen than the tremor sub-score. While there was no significant difference in the correlation coefficients of the correlations with axial and LAR MDS-UPDRS-III sub-scores ($t_{axial\_vs\_LAR} = -1.19$, $p_{axial\_vs\_LAR} = 0.12$), both differed significantly from the correlation coefficient derived with the tremor sub-score. ($t_{axial\_vs\_tremor} = -4.73$, $p_{axial\_vs\_tremor} < 0.001$, $t_{LAR\_vs\_tremor} = -3.74$, $p_{LAR\_vs\_tremor} < 0.001$).

***axial** MDS-UPDRS-III sub-score * mean putaminal dopamine transporter signal*
(τ =-0.27, p <0.001)

vs.

***LAR** MDS-UPDRS-III sub-score * mean putaminal dopamine transporter signal*
(τ = -0.2, p < 0.001)

vs.

***Tremor** MDS-UPDRS-III sub-score * mean putaminal dopamine transporter signal*
(τ = 0.06, p = 0.30)

Further, the correlation analysis between the tremor sub-score and the putaminal dopamine transporter signal did not reach the significance threshold. This supports previous evidence that the tremor-related items of the MDS-UPDRS-III score are not a direct cause of the dopaminergic cell loss.

3) The regression model predicting the less affected axial LAR MDS-UPDRS-III sub-score by the contralateral putaminal dopamine transporter signal showed a better model fit compared to the one modelling the more affected sub-score.

less affected *axial LAR sub-score ~ contralateral putaminal signal, age, sex*

($F(3,147)=11.4$, $p<0.001$, $r=.43$, $r^2=.19$)

vs.

more affected *axial LAR sub-score ~ contralateral putaminal signal, age, sex*

($F(3,147)=8.0$, $p<0.001$, $r=.38$, $r^2=.14$)

Both regression models were significant, but a better model fit was observed for the model predicting the less affected body side. Closer investigations revealed a flooring effect regarding the correlation between the more affected axial LAR sub-score and contralateral putaminal dopamine transporter signal (Supplementary Figure 1). Ceiling and flooring effects are both critical for the application of the residual approach, given the range of the predictor is limited (Elman *et al.*, 2022). The absolute difference in model fit regarding r^2 was 0.05 ($r_{\text{less affected}} - r_{\text{more affected}}$ = 0.19-0.14) or 26% (0.19/100*0.05) when considering $r^2=0.19$ as the best possible model fit. Even when applying a model-independent benchmark for model comparison like 25% ($r^2=0.25$, $r=0.5$), which characterizes highly predictive

variables in disease-related biological systems, the observed difference in r^2 of 0.05 accounts for 1/5 = 20% of the maximal assumed explanatory power.

In summary, considering the difference in model fit and the described flooring effect, the closest association between dopamine transporter signal and MDS-UPDRS-III score was achieved with the less affected MDS-UPDRS-III sub-score containing axial and LAR items and the contralateral putaminal dopamine transporter signal.

Association between resilience and daily physical activity

Seven partial Spearman correlation analyses between Physical Activity Scales of the Elderly (PASE) of different assessment dates (every full year from year 1 till year 7) and the baseline resilience estimates were performed (Supplementary Figure 2). All showed the same trend, namely higher resilience estimates (negative residual values) being associated with greater physical activity scores. While this significant association validated the derived resilience estimates for the year one correlation, significant correlations at later time points indicate that the resilience estimates might have a predictive value for future physical activity levels.

Linear mixed modelling to assess longitudinal resilience effects

Linear mixed model, including cognitive function and medication information

Including the levodopa equivalent daily dose (LEDD) and MoCA score did not change the results of the time*residual category interaction term or any other fixed parameter in the mixed model analysis. While the baseline MoCA score did not significantly contribute to the models ($p>0.05$), the LEDD was significant when predicting the total MDS-UPDRS-III score or the more affected axial limb-akinetic-rigid sub-score (for test statistic and p-values see Supplementary Table 2).

Survival analysis - Time until onset of levodopa-induced dyskinesia

Six Kaplan-Meier survival analyses, regarding the time until onset of levodopa-induced dyskinesia based on either MDS-UPDRS-III or -IV (motor complication) assessments over three different time intervals, were performed. The analyses revealed a trend of the curves of the higher resilience group laying above those of the low resilience group, indicating that high resilience patients experience longer intervals until the onset of levodopa-induced dyskinesia. However, the difference in survival time did not reach significance ($p<0.05$). Different time intervals (84, 102, 156 months), information sources (MDS-UPDRS-III or -IV score), or residual levels (residual split at 0SD, +/-0.5SD, +/-1SD) did not influence the result of the analyses (for survival curves and associated test statistics, see Supplementary Figure 4).

Methods

Correlations between dopamine transporter signals and MDS-UPDRS-III sub-scores

The hierarchical hypothesis testing, as illustrated in Figure 7 in the manuscript, is based on Williams tests or the comparison of r^2 values from evaluated regression models. For the Wiliams tests, Kendall's tau b was transformed in Pearson's r by using Kendall's formula[1]:

$$r=\sin(0.5\pi\tau)$$

All results regarding the Williams test are reported with one-tailed p-values given the clearly defined hypotheses.

1. Hypothesis

The first hypothesis claims that the putaminal dopamine transporter signal is closer associated with the MDS-UPDRS-III score than the dopamine transporter signal of the caudate nucleus. To test this hypothesis, the correlation coefficients of the correlations between the mean dopamine transporter signal of the putamen, or caudate nucleus with the entire MDS-UPDRS-III score, were compared.

*mean **putaminal** dopamine transporter signal * MDS-UPDRS-III score*

***vs**.*

*mean **caudate** nucleus dopamine transporter signal * MDS-UPDRS-III score*

2. Hypothesis

The second hypothesis claims that the axial and LAR MDS-UPDRS-III sub-scores are closer associated with the dopamine transporter signal of the putamen than the tremor sub-score. To test this hypothesis, the correlation coefficients of the correlation between 1) axial, or 2) LAR, or 3) tremor sub-scores with the dopamine transporter signal of the mean putamen were compared.

axial *MDS-UPDRS-III sub-score * mean putaminal dopamine transporter signal*

vs.

LAR *MDS-UPDRS-III sub-score * mean putaminal dopamine transporter signal*

vs.

tremor *MDS-UPDRS-III sub-score * mean putaminal dopamine transporter signal*

3. Hypothesis:

The third hypothesis considers the laterality of the dopaminergic degeneration and laterality of symptom onset. Considering the crossing of the fibre bundles, the more affected bodyside should be located contralateral to the more affected hemisphere, while in this case, the less affected hemisphere should be located ipsilateral. Considering this anatomical basis and previous studies, anatomically ipsilateral correlations were considered to be less meaningful in a biological sense than anatomically contralateral ones. Hypothesis three claims that the less affected bodyside and contralateral hemisphere are more closely associated than the more affected bodyside and contralateral hemisphere. To test this hypothesis, we compared two regression models, predicting either the less or more affected MDS-UPDRS-III sub-score of axial and limb-akinetic-rigid items by the respective contralateral

putaminal dopamine transporter signal. Additionally, both models included age and sex as covariables.

__less affected__ axial LAR MDS-UPDRS-III sub-score ~

contralateral putaminal dopamine transporter signal, age, sex

vs.

__more affected__ axial LAR MDS-UPDRS-III sub-score ~

contralateral putaminal dopamine transporter signal, age, sex

Linear mixed modelling to assess longitudinal resilience effects

Linear mixed model, including cognitive scores and medication information

We addressed potential confounding effects of cognitive status and medication by incorporating LEDD and MoCA scores as continuous covariates in our mixed model analyses. The LEDD information was computed at each time point with an available MDS-UPDRS-III score. First, we filtered the "LEDD_concomitant_Medication_Log" file to obtain the prescribed medicines at the respective MDS-UPDRS-III assessment dates. Then, we added all levodopa doses and agonists multiplied by the respective conversion factors. Next, COMT inhibitors were accounted for by multiplying the daily dose by the inhibition factor and adding it to the respective dose. If missing data (e.g., unavailable prescription start dates) prevented an exact calculation of the LEDD, we treated the data at this time point as missing. Finally, like the MDS-UPDRS-III data, we smoothed the LEDD data by averaging scores over one year.

Survival analysis - Time until onset of levodopa-induced dyskinesia

To assess potential long-term effects of different resilience levels (0, +/- 0.5SD and +/- 1SD) more comprehensively, we performed Kaplan-Maier survival analyses to identify the resilience level-dependent time until the onset of levodopa-induced dyskinesia. The occurrence of levodopa-induced dyskinesia was assessed by means of the MDS-UPDRS-III questionnaire (Question: “Were dyskinesias present?”) and the MDS-UPDRS-IV questionnaire item 4.1 (Question: “Time spent with dyskinesias?”). We assessed both questionnaires independently to consider potential differences between self-reported (MDS-UPDRS-IV) and rater-dependent (MDS-UPDRS-III) information. Analyses were performed at three different time intervals (84, 102, 156 months). For data availability at the different time points, see Supplementary Table 8.

The following open-source software versions and packages were used for the statistical analysis:

RStudio version 1.3.959 (R: A language and environment for statistical computing, 2020; R Core Team, R Foundation for Statistical Computing, Vienna, Austria. (https://www.R-project.org/), packages MASS[2] (version 7.3.51.5) , ppcor[3] (version 1.1) and psych[4] (version 1.9.12.31).

Python version 3.8 (Van Rossum, G., & Drake Jr, F. L. (1995). Python reference manual. Centrum voor Wiskunde en Informatica Amsterdam) in form of a jupyter notebook[5] (version 6.1.4), packages numpy[6] (1.19.5), matplotlib[7] (version 3.2.1), seaborn[8] (version 0.10.1), pandas[9] (version 1.3.5), and scipy.stats[10] (version 1.4.1).

References

1 Walker, D. A. Converting Kendall's Tau For Correlational Or MEta-Analytic Analysis. *Journal of Modern Applied Statistical Methods* **2**, 525-530 (2003).

2 W. N. Venables, B. D. R. *Modern Applied Statistics with S*. fourth edn, (Springer, 2002).

3 Kim, S. ppcor: An R Package for a Fast Calculation to Semi-partial Correlation Coefficients. *Commun Stat Appl Methods* **22**, 665-674 (2015). https://doi.org:10.5351/CSAM.2015.22.6.665

4 Revelle, W. psych: Procedures for Psychological, Psychometric, and Personality Research (2020).

5 Thomas Kluyver, B. R.-K., Fernando Pérez et al. Jupyter Notebooks -- a publishing format for reproducible computational workflows. *IOS Press*, 87 - 90 (2016).

6 Van Der Walt, S. a. C., S Chris and Varoquaux, Gael. The NumPy Array: A Structure for Efficient Numerical Computation. *Computing in Science & Engineering* **13**, 22 (2011).

7 Hunter, J. D. Matplotlib is a 2D graphics package used for Python for application development, interactive scripting,and publication-quality image generation across user interfaces and operating systems. *Computing in Science & Engineering* **9** (2007). https://doi.org:10.1109/MCSE.2007.55

8 Waskom, M. L. seaborn: statistical data visualiszation. *Journal of the Opaen Source Software* **6**, 3021 (2021). https://doi.org:10.21105/joss.03021

9 McKinney, W. *Proceedings of the 9th Python in Science Conference -Data Structures for Statistical Computing in Python-*. 51-56 (2010).

10 Virtanen, P. *et al.* SciPy 1.0: fundamental algorithms for scientific computing in Python. *Nat Methods* **17**, 261-272 (2020). https://doi.org:10.1038/s41592-019-0686-2

Supplementary figure and figure legends

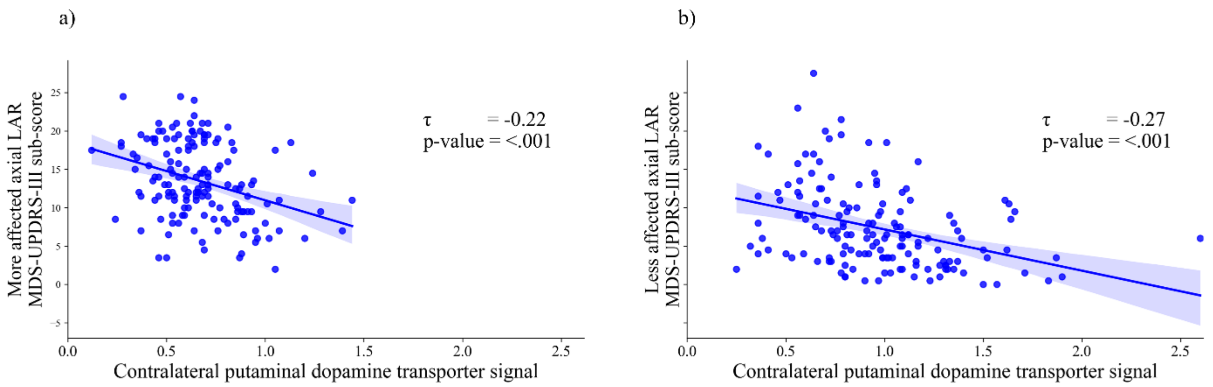

Supplementary Figure 1 Visualization of flooring effects observed in correlations with the more affected putaminal dopamine transporter signal

Kendall tau b correlations between the a) more affected axial LAR MDS-UPDRS-III sub-score or; b) the less affected axial LAR MDS-UPDRS-III sub-score and respective contralateral putaminal dopamine transporter signal. Visualization revealed flooring effects in correlations with the more affected putaminal dopamine transporter signal. The plots display 95% confidence intervals as error bars, estimated using bootstrapping with 1000 iterations. LAR = limb-akinetic-rigid; MDS-UPDRS-III = Movement Disorder Society – Unified-Parkinson's-Disease-Rating-Scale motor-score

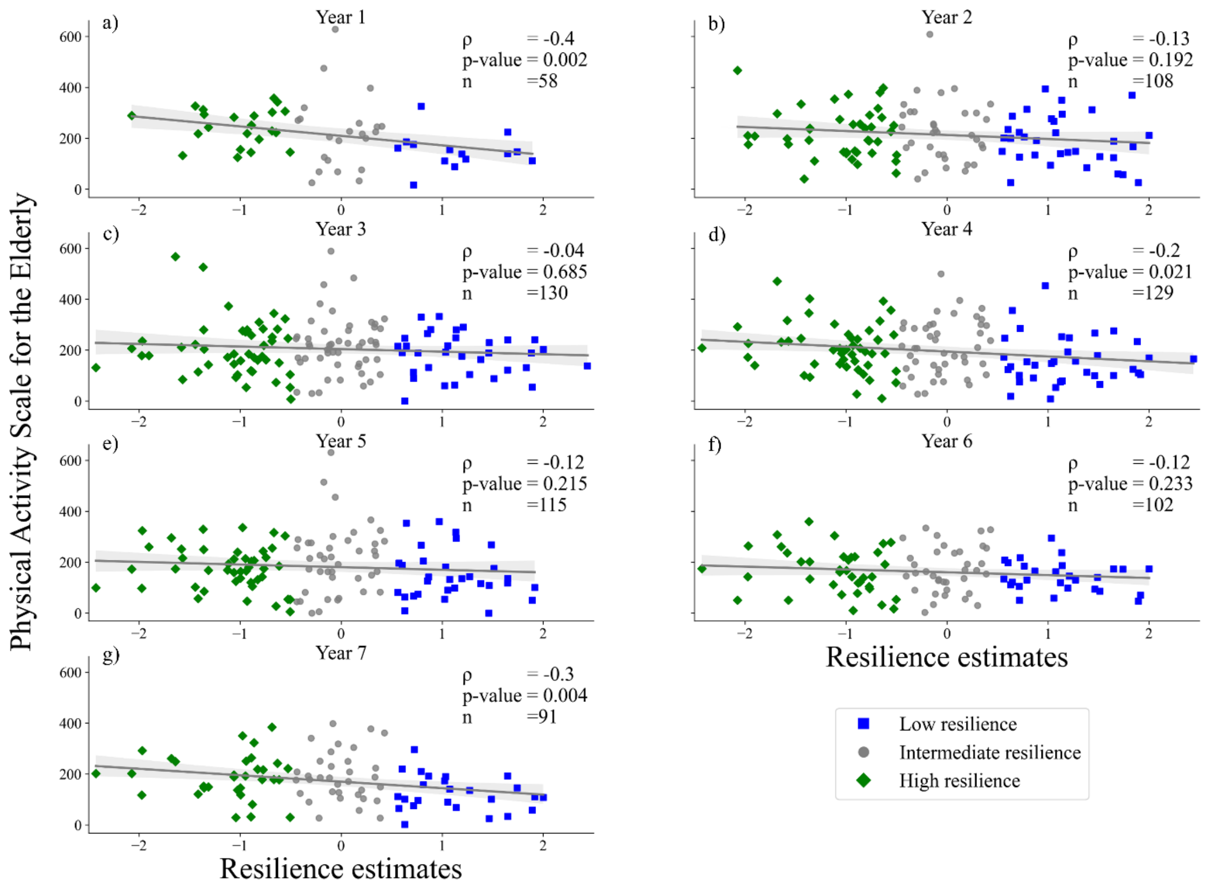

Supplementary Figure 2 Correlation between baseline resilience values and daily physical activity from year one to seven

Partial Spearman correlation between the baseline resilience estimates and the Physical Activity Scale for the Elderly score at year one till year seven follow-up, corrected for age and sex. Low, intermediate, and high resilience patients are indicated by blue-filled squares, grey-filled circles, and green-filled diamonds, respectively. The plots display 95% confidence intervals as error bars, estimated using bootstrapping with 1000 iterations.

gre

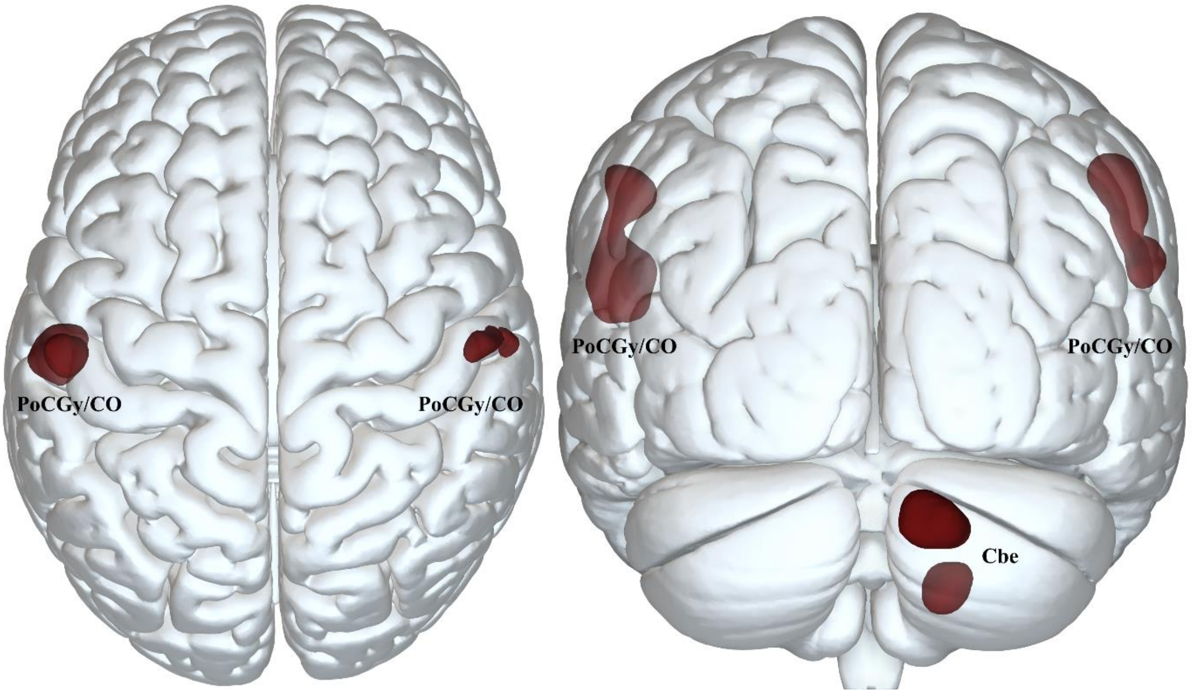

Supplementary Figure 3 - Voxel-wise grey matter volume group comparison considering dominant affected side as covariate

Adding the dominant affected side as a covariate yielded clusters of higher grey matter volume in high-resilience patients in the same brain regions as in the previous analyses (i.e. postcentral gyrus (PoCGy), central operculum (CO), and exterior cerebellum (Cbe)). Notably, the cluster encompassing the left postcentral gyrus exhibited an increased extent, and an additional cluster emerged rostrally and ventrally to the pre-existing one in the right exterior cerebellum. No significant clusters of increased grey matter volume were found using the reversed contrast. All clusters shown here were significant at cluster-level after FWE-correction ($p<0.05$) with an initial p value set at $p<0.001$.

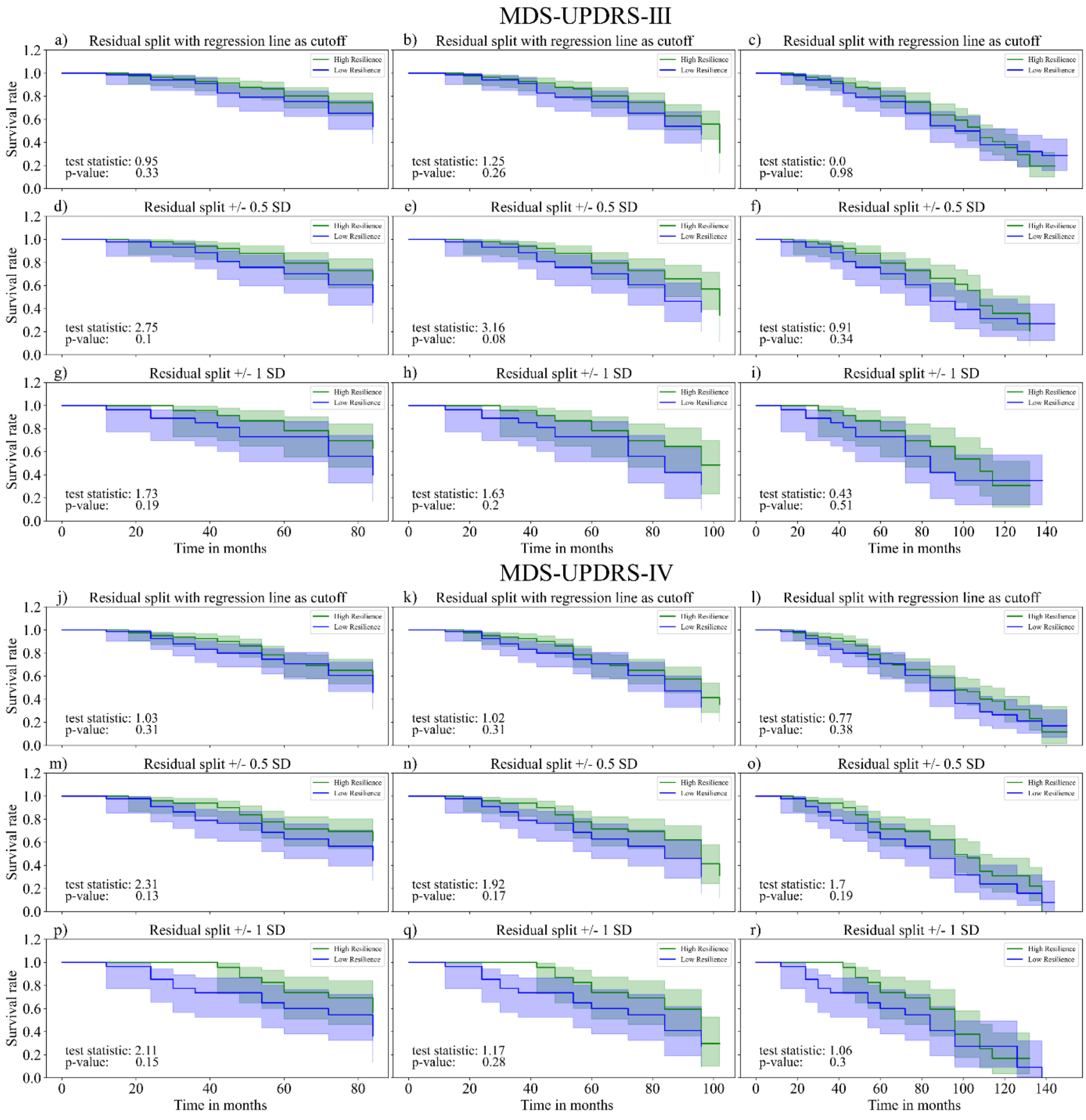

Supplementary Figure 4 - Kaplan Meier survival curves for resilience level-dependent time until onset of levodopa-induced dyskinesia

We explored three residual splits (0, +/-0.5, +/-1 SD from top to bottom) and three time intervals (left to right: 84 (7), 102 (8.5), and 156 (13) months (years)), utilizing levodopa-induced dyskinesia information extracted from the MDS-UPDRS-III (upper nine plots (a-i)) and IV questionnaires (bottom nine plots (j-r)). However, none of the analyses reached statistical significance (α=0.05). The plots display 95% confidence

intervals as error bars. MDS-UPDRS-III = Movement Disorder Society - Unified-Parkinson’s-Disease-Rating-Scale motor-score, IV = motor complications

Supplementary tables and table legends

Supplementary Table 1 - Predictor ß-coefficients and p-values of the MDS-UPDRS-III-OFF score linear mixed model analyses

LINEAR MIXED MODEL ANALYSIS OF RESILIENCE DEPENDENT LONGITUDINAL MDS-UPDRS-III SCORE DECLINE						
**PREDICTORS**	More affected sub-score		MDS-UPDRS-III score		Less affected sub-score	
**FIXED EFFECTS**	ß	p-value 95% CI	ß	p-value 95% CI	ß	p-value 95% CI
**AGE**	0.01	0.59 -0.02:0.04	0.04	0.03 0.00:0.08	0.04	0.01 0.01:0.07
**SEX (0 = FEMALE, 1 = MALE)**						
**0**	-0.27	0.04 -0.52:-0.02	-0.39	0.01 -0.70:-0.09	-0.44	<0.001 -0.69:-0.20
**BASELINE DOPAMINE TRANSPORTER SIGNAL**	-1.5	<0.001 -1.98:-0.93	-0.92	<0.001 -1.37:-0.47	-1.27	<0.001 -1.63:-0.91
**LINEAR TIME (YEARS)**	0.19	<0.001 0.11:0.27	0.33	<0.001 0.23:0.44	0.28	<0.001 0.19:0.37
**QUADRATIC TIME (YEARS)**	-0.02	<0.001 -0.02:-0.01	-0.03	<.001 -0.04:-0.02	-0.02	<0.001 -0.03:-0.01
**RESIDUAL CATEGORY (0= INTERMEDIATE,1= HIGH, 2= LOW RESILIENCE)**						
**0**	-0.51	=0.001 -0.81:-0.20	-0.7	<0.001 -1.07:-0.32	-0.98	<0.001 -1.28:-0.68
**1**	-0.98	<.001 -1.29:-0.67	-1.41	<.001 -1.79:-1.03	-1.85	<.001 -2.16:-1.55
**RESIDUAL CATEGORY*TIME**						
**0**	0	0.91 -0.07:0.08	0.03	0.52 -0.07:0.13	0.06	0.13 -0.02:0.14
**1**	0.10	0.01 0.03:0.18	0.13	0.01 0.04:0.23	0.16	<0.001 0.08:0.24
**RANDOM EFFECTS**	ß	p-value	ß	p-value	ß	p-value
**SUBJECT (INTERCEPT)**	0.25	<0.001	0.25	<0.001	0.18	<0.001
**TIME (SLOPE)**	0.01	<0.001	0.02	<0.001	0.01	<0.001
**COVARIANCE (INTERCEPT*SLOPE)**	0.00	0.98	0.01	0.66	0.01	0.40
**YEARS OF BENEFIT**						
	Years	CI	Years	CI	Years	CI
	11.4	1.1 : 21.7	12.2	0.2 : 24.3	12.2	2.9 : 21.5

The table provides the unstandardized ß-coefficients, p-values and 95% confidence intervals (CI) for the three linear mixed models. The models were set up to estimate the resilience level dependent disease trajectories of the more and less affected axial LAR MDS-UPDRS-III-OFF sub-score as well as the total MDS-UPDRS-III-OFF score over seven years. On the upper part the included fixed effects are shown, while the middle part displays the estimates of the unstructured covariance matrix of the two random effects. Further, the bottom part shows the interpolation results for the years it would take for patients with high resilience to be on par with the motor performance levels of patients with low resilience. MDS-UPDRS-III-OFF = Movement Disorder Society Unified-Parkinson's-Disease-Rating-Scale motor-score off-medication

Supplementary Table 2 - Predictor ß-coefficients and p-values of the MDS-UPDRS-III-OFF score linear mixed model analyses including cognitive scores and medication as covariates

LINEAR MIXED MODEL ANALYSIS OF RESILIENCE DEPENDENT LONGITUDINAL MDS-UPDRS-III SCORE DECLINE						
**PREDICTORS**	More affected sub-score		MDS-UPDRS-III score		Less affected sub-score	
**FIXED EFFECTS**	ß	p-value<br>95% CI	ß	p-value<br>95% CI	ß	p-value<br>95% CI
**AGE**	0.01	0.67<br>-0.02 : 0.04	0.04	0.04<br>0.00: 0.08	0.04	0.01<br>0.01 : 0.07
**SEX (0 = FEMALE, 1 = MALE)**						
**0**	-0.31	0.03<br>-0.57 : -0.04	-0.43	0.01<br>-0.74:-0.12	-0.48	<0.001<br>-0.74 : -0.22
**BASELINE DOPAMINE TRANSPORTER SIGNAL**	-1.41	<0.001<br>-1.97 : -0.85	-0.93	<0.001<br>-1.38 : -0.47	-1.26	<0.001<br>-1.63 : -0.88
**LINEAR TIME (YEARS)**	0.23	<0.001<br>0.14 : 0.32	0.38	<0.001<br>0.27 : 0.49	0.30	<0.001<br>0.21 : 0.39
**QUADRATIC TIME (YEARS)**	-0.02	<0.001<br>-0.02 : -0.01	-0.03	<0.001<br>-0.04 : -0.02	-0.02	<0.001<br>-0.03 : -0.01
**MoCA**	0.02	0.52<br>-0.04 : 0.07	0.02	0.58<br>-0.05 : 0.08	0.04	0.16<br>-0.02 : 0.09
**LEVODOPA EQUIVALENT DAILY DOSE**	-0.27	0.01<br>-0.47 : -0.07	-0.35	0.01<br>-0.61 : -0.10	-0.16	0.14<br>-0.37: 0.05
**RESIDUAL CATEGORY (0= INTERMEDIATE,1= HIGH, 2= LOW RESILIENCE)**						
**0**	-0.54	=0.001<br>-0.86 : -0.22	-0.74	<0.001<br>-1.12 : 0.36	-1.00	<0.001<br>-1.31 : -0.69
**1**	-1.01	<0.001<br>-1.34 : -0.68	-1.44	<0.001<br>-1.83 : 1.06	-1.87	<0.001<br>-2.18 : -1.55
**RESIDUAL CATEGORY*TIME**						
**0**	0.01	0.86<br>-0.07 : 0.09	0.04	0.46<br>-0.06 : 0.14	0.07	0.12<br>-0.02 : 0.15
**1**	0.10	0.02<br>0.02 : 0.18	0.12	0.02<br>0.02 : 0.23	0.16	<0.001<br>0.08 : 0.24
**RANDOM EFFECTS**	ß	p-value	ß	p-value	ß	p-value
**SUBJECT (INTERCEPT)**	0.20	<0.001	0.26	<0.001	0.17	<0.001
**TIME (SLOPE)**	0.01	0.48	0.00	0.78	0.01	0.22
**COVARIANCE (INTERCEPT*SLOPE)**	0.01	<0.001	0.02	<0.001	0.01	<0.001

The table provides the unstandardized ß-coefficients, p-values and 95% confidence intervals (CI) for the three linear mixed models. The models were set up to estimate the resilience level-dependent disease trajectories of the more and less affected axial LAR MDS-UPDRS-III-OFF sub-scores as well as the total MDS-UPDRS-III-OFF score over seven years. In addition to the information provided in Supplementary Table 1, analyses listed in this table included the baseline Montreal Cognitive Assessment (MoCA) score and longitudinal levodopa equivalent daily dose as covariates. On the upper part the included fixed effects are shown, while the lower part displays the estimates of the unstructured covariance matrix of the two random effects. CI = Confidence Interval, MDS-UPDRS-III-OFF = Movement Disorder Society – Unified-Parkinson's-Disease-Rating-Scale motor-score off-medication

Supplementary Table 3 - Predictor ß-coefficients and p-values of the dopamine transporter linear mixed model analyses

LINEAR MIXED MODEL ANALYSIS OF MOTOR RSILIENCE DEPENDENT LONGITUDINAL DOPAMINE TRANSPORTER DECLINE						
**PREDICTORS**	More affected putaminal signal		Mean putaminal signal		Less affected putaminal signal	
**FIXED EFFECTS**	ß	p-value<br>95% CI	ß	p-value<br>95% CI	ß	p-value<br>95% CI
**AGE**	0.00	0.89<br>-0.01:0.01	0.00	0.86<br>-0.01:0.01	0.00	0.67<br>-0.01:0.01
**SEX (0 = FEMALE, 1 = MALE)**						
**0**	0.04	0.28<br>-0.03:0.10	0.06	0.11<br>-0.01:0.14	0.07	0.12<br>-0.02:0.01
**LINEAR TIME (YEARS)**	-0.08	<0.001<br>-0.11:-0.05	-0.10	<0.001<br>-0.13:-0.08	-0.13	<0.001<br>-0.16:-0.09
**QUADRATIC TIME (YEARS)**	0.01	<0.001<br>0.00:0.02	0.01	<0.001<br>0.01:0.02	0.01	<0.001<br>0.01:0.02
**RESIDUAL CATEGORY (0= INTERMEDIATE,1= HIGH, 2= LOW RESILIENCE)**						
**0**	0.01	0.81<br>-0.08:0.11	0.00	0.97<br>-0.12:0.11	-0.02	0.80<br>-0.17:0.13
**1**	0.03	0.58<br>-0.07:0.12	0.03	0.62<br>-0.08:0.14	0.03	0.70<br>-0.12:0.18
**RESIDUAL CATEGORY*TIME**						
**0**	0.00	0.75<br>-0.02:0.02	0.00	0.91<br>-0.02:0.02	0.00	0.93<br>-0.03:0.02
**1**	0.00	0.94<br>-0.02:0.02	-0.01	0.62<br>-0.02:0.01	-0.01	0.51<br>-0.03:0.02
**RANDOM EFFECTS**	ß	p-value	ß	p-value	ß	p-value
**SUBJECT (INTERCEPT)**	0.03	<0.001	0.05	<0.001	0.09	<0.001
**TIME (SLOPE)**	0.00	0.24	0.00	0.01	0.00	0.01
**COVARIANCE (INTERCEPT*SLOPE)**	0.00	0.02	0.00	<0.001	-0.01	<0.001

The table provides the unstandardized ß-coefficients, p-values, and 95% confidence intervals (CI) for the three linear mixed models. These models were set up to estimate the resilience level-dependent, four-year longitudinal decline of putaminal dopamine transporter availability. The analysis considered both contralateral and ipsilateral hemispheres, as well as the combined data across both hemispheres. On the upper part the included fixed effects are shown, while the lower part displays the estimates of the unstructured covariance matrix of the two random effects.

Supplementary Table 4 - Subject IDs

**Cohorts based on the residual split at the regression line**	
High resilience (residuals < 0 SD)	3001, 3603, 3012, 3606, 3021, 3622, 3056, 3638, 3061, 3661, 3062, 3664, 3066, 3666, 3078, 3700, 3086, 3710, 3113, 3775, 3118, 3776, 3122, 3808, 3124, 3815, 3126, 3819, 3128, 3822, 3150, 3823, 3173, 3824, 3174, 3825, 3175, 3830, 3176, 3832, 3179, 3838, 3184, 3914, 3185, 3954, 3186, 3963, 3305, 4034, 3307, 4037, 3309, 4056, 3322, 4057, 3327, 4099, 3366, 4112, 3373, 4117, 3377, 3385, 3415, 3417, 3451, 3459, 3469, 3470, 3472, 3473, 3482, 3507, 3522, 3532, 3567, 3586, 3587, 3591, 3592, 3593
Low resilience (residuals >0 SD)	3003, 3972, 3018, 4011, 3024, 4012, 3052, 4013, 3077, 4019, 3083, 4020, 3088, 4035, 3089, 4051, 3102, 4054, 3111, 4058, 3116, 4071, 3119, 4072, 3129, 4074, 3166, 4093, 3168, 4098, 3182, 4121, 3352, 4123, 3392, 4136, 3409, 3418, 3434, 3435, 3440, 3443, 3454, 3461, 3476, 3504, 3530, 3540, 3552, 3556, 3557, 3558, 3574, 3575, 3601, 3607, 3621, 3629, 3630, 3632, 3752, 3763, 3770, 3777, 3778, 3789, 3866, 3910, 3911
**Cohorts based on the residual split excluding residuals within +/- 0.5 standard deviations around the regression line**	
High resilience (residuals < -0.5 SD)	3001, 3056, 3062, 3066, 3078, 3086, 3113, 3122, 3124, 3128, 3173, 3176, 3184, 3185, 3307, 3322, 3327, 3373, 3377, 3417, 3459, 3470, 3472, 3473, 3482, 3507, 3586, 3587, 3591, 3593, 3661, 3664, 3666, 3700, 3776, 3815, 3819, 3822, 3823, 3825, 3830, 3832, 3838, 3914, 3954, 3963, 4034, 4037, 4112, 4117
Low resilience (residuals > 0.5 SD)	3003, 3077, 3083, 3088, 3089, 3111, 3116, 3129, 3166, 3168, 3182, 3352, 3392, 3434, 3443, 3461, 3504, 3530, 3540, 3557, 3558, 3574, 3575, 3601, 3607, 3629, 3763, 3770, 3777, 3778, 3789, 3866, 3972, 4011, 4012, 4013, 4020, 4054, 4058, 4071, 4072, 4074, 4093, 4121, 4123
**Cohorts for the linear mixed model of the Movement Disorder Society Unified-Parkinson's-Disease-Rating-Scale motor-score**	
High resilience (residuals < -0.5 SD)	3001, 3056, 3062, 3066, 3078, 3122, 3124, 3128, 3173, 3176, 3184, 3322, 3373, 3377, 3417, 3459, 3470, 3472, 3776, 3815, 3819, 3822, 3823, 3830, 3954
Intermediate resilience (0.5 SD > residuals >-0.5 SD)	3012, 3018, 3021, 3052, 3119, 3126, 3305, 3309, 3409, 3415, 3418, 3435, 3451, 3454, 3469, 3522, 3532, 3552, 3556, 3603, 3622, 3808, 4051, 4056, 4057, 4098, 4099
Low resilience (residuals > 0.5 SD)	3077, 3083, 3111, 3168, 3434, 3443, 3461, 3557, 3601, 3607, 3629, 3777, 4011, 4054, 4058, 4071, 4072, 4074
**Cohorts for the linear mixed model of the Dopamine transporter signal**	
High resilience (residuals < -0.5 SD)	3001, 3056, 3062, 3066, 3078, 3086, 3122, 3124, 3128, 3173, 3176, 3184, 3185, 3307, 3327, 3373, 3377, 3417, 3459, 3470, 3472, 3473, 3482, 3587, 3591, 3593, 3661, 3664, 3666, 3776, 3815, 3819, 3822, 3823, 3825, 3830, 3832, 3838, 3954, 4034, 4117
Intermediate (0.5 SD > residuals >-0.5 SD)	3012, 3061, 3118, 3126, 3174, 3175, 3179, 3186, 3309, 3366, 3385, 3415, 3451, 3469, 3522, 3532, 3567, 3592, 3603, 3622, 3710, 3775, 3808, 3824, 4056, 4057, 4099, 3018, 3052, 3102, 3119, 3409, 3418, 3435, 3454, 3552, 3556, 3752, 4019, 4035, 4051, 4098
Low resilience (residuals>0.5 SD)	3003, 3077, 3083, 3088, 3089, 3116, 3168, 3182, 3352, 3392, 3434, 3443, 3461, 3504, 3530, 3540, 3557, 3607, 3629, 3763, 3770, 3777, 3778, 3789, 4011, 4012, 4020, 4054, 4058, 4071, 4093, 4123

Subject IDs from the Parkinson's Progression Markers Initiative database of the different cohorts.SD = Standard Deviation

Supplementary Table 5 - Physical Activity Scale for the Elderly score availability

Assessment time point	Months	Availability/ entire cohort [%]	High resilience [%]	Low resilience [%]
BL	0	5,3	2.0	8.9
V01	3	0.7	0.0	0.0
V04	12	38.4	14.6	31.1
V05	18	1.3	0.0	2.2
V06	24	71.5	82.0	73.3
V08	30	86.1	94.0	73.3
V10	36	85.4	96.0	80.0
V12	48	76.2	90.0	66.7
V13	60	67.5	76.0	62.2
V14	72	60.3	64.0	55.6
V15	84	19.8	22.0	31.1
V16	96	3.3	0.0	2.2

Physical Activity Scale for the Elderly availability at different assessment time points for the entire Parkinson's disease cohort as well as for the high and low resilience groups, respectively.

Supplementary Table 6 - Demographic and clinical characteristics of patients with Parkinson's disease

		ENTIRE PARKINSON'S DISEASE COHORT		GROUP COMPARISON -HIGH VS. LOW RESILIENCE-		
		**Characteristics**	**Average +/- SD**	**High resilience Average +/- SD**	**Low resilience Average +/- SD**	**p-value***
**DEMOGRAPHICS**		Number	151	82	69	
		Sex (M/F)	89 / 62	49/33	40/29	0.96
		Age, y	58.7 ± 4.5	58.7 ± 4.2	58.8 ± 4.7	0.43
		Education, y	15.6 ± 3.0	15.6 ± 3.4	15.7 ± 2.4	0.45
		MoCA	27.3 ± 2.0	27.5 ± 2.1	27.1 ± 1.9	0.08
		TIV	1480.8 ± 149.6	1496.2 ± 145.8	1462.5 ± 152.1	0.07
		Handedness (left/ **right**/ mixed)	19 / **128** / 4	8 / **73** / 1	11 / **55** / 3	0.23
		More affected side (left / **right** / no)	65 / **85** / 1	35 / **47** / 0	30 / **38** / 1	0.54
**DOPAMINE TRANSPORTER (SBR) HEMISPHERE**	Mean	Caudate	2.00 ± 0.55	2.01 ± 0.50	2.00 ± 0.59	0.48
		**Putamen**	**0.82 ± 0.27**	**0.83 ± 0.25**	**0.80 ± 0.29**	**0.13**
		Striatum	1.41 ± 0.38	1.42 ± 0.35	1.40 ± 0.42	0.28
	More affected	Caudate	1.83 ± 0.53	1.84 ± 0.51	1.81 ± 0.55	0.49
		**Putamen**	**0.67 ± 0.22**	**0.69 ± 0.21**	**0.65 ± 0.24**	**0.13**
		Striatum	1.25 ± 0.35	1.26 ± 0.33	1.23 ± 0.37	0.39
	Less affected	Caudate	2.18 ± 0.60	2.18 ± 0.53	2.18 ± 0.67	0.43
		**Putamen**	**0.96 ± 0.37**	**0.97 ± 0.33**	**0.96 ± 0.40**	**0.17**
		Striatum	1.57 ± 0.45	1.58 ± 0.40	1.57 ± 0.51	0.30
**MDS-UPDRS-III BODYSIDE**	total	Both	20.1 ± 7.8	15.3 ± 5.1	25.8 ± 6.5	< 0.001
		**Less affected**	**9.2 ± 5.6**	**5.6 ± 2.9**	**13.4 ± 5.1**	**< 0.001**
		More affected	17.1 ± 5.3	14.3 ± 4.5	20.4 ± 4.1	< 0.001
	Limb-akinetic-rigid	Both	13.0 ± 6.4	9.2 ± 3.8	17.6 ± 6.0	< 0.001
		**Less affected**	**4.4 ± 4.2**	**1.8 ± 1.6**	**7.5 ± 4.4**	**< 0.001**
		More affected	10.5 ± 4.0	8.6 ± 3.4	12.8 ± 3.4	< 0.001
	Tremor	Both	4.1 ± 2.7	4.1 ± 2.5	4.1 ± 2.8	0.38
		**Less affected**	**1.8 ± 1.4**	**1.8 ± 1.3**	**1.8 ± 1.6**	**0.43**
		More affected	3.6 ± 2.4	3.7 ± 2.3	3.6 ± 2.5	0.41
		Axial	3.0 ± 1.8	2.0 ± 1.2	4.1 ± 1.7	< 0.001

Patients' characteristics in the entire Parkinson's disease cohort are summarized as mean ± standard deviation. MDS-UPDRS-III=Movement Disorder Society – Unified-Parkinson's-Disease-Rating-Scale motor-score; MoCA = Montreal-Cognitive-Assessment-Test; SD = standard deviation; SBR= Specific Binding Ratio; TIV=Total Intracranial Volume,

*Patients with low (>0 residual value) or high resilience (<0 residual value) were identified and compared using Mann-Whitney-U for continuous and chi-square tests for categorical variables.

Supplementary Table 7 - Characteristics of the MDS-UPDRS-III-OFF linear mixed model cohort

LMM MDS-UPDRS-III COHORT -7 YEAR FOLLOW-UP-			GROUP COMPARISON -HIGH VS. INTERMEDIATE VS. LOW RESILIENCE-			
**DEMOGRAPHICS**	**Characteristics**	**Average +/- SD**	**High resilience**	**Intermediate resilience**	**Low resilience**	**p-value**
	Number	70	25	27	18	
	Sex (m/w)	46/24	15/10	20/7	11/7	0.50
	Age	59.5± 4.0	59.1 ± 3.9	59.8 ± 3.9	59.5 ± 4.2	0.78

Patient characteristics of the entire linear mixed model cohort, for whom 7-years follow-up information of MDS-UPDRS-III-OFF scores were available, as well as the group-specific information of patients with high, intermediate and low resilience. The last column indicates if there are significant between-group differences. LMM = linear mixed model, MDS-UPDRS-III-OFF = Movement Disorder Society - Unified-Parkinson's-Disease-Rating-Scale motor-score off-medication, SD = standard deviation

Supplementary Table 8 - Kaplan Meier survival curve analysis MDS-UPDRS-III and -IV data availability

**Event ID**	**Months**	***MDS-UPDRS-III* Availability in % from n subjects**			***MDS-UPDRS-IV* Availability in % from n subjects**		
		Entire cohort n=151	High resilience *SD = 0 / 0.5 / 1* n = 89 / 50 / 23	Low resilience *SD = 0 / 0.5 / 1* n = 69 / 45 / 28	Entire cohort n=151	High resilience *SD = 0 / 0.5 / 1* n = 89 / 50 / 23	Low resilience *SD = 0 / 0.5 / 1* n = 69 / 45 / 28
**SC**	-1.5	100	100 / 100 / 100	100 / 100 / 100	0	0 / 0 / 0	0 / 0 / 0
**BL**	0	100	100 / 100 / 100	100 / 100 / 100	0	0 / 0 / 0	0 / 0 / 0
**V01**	3	97	98 / 98 / 96	97 / 96 / 100	6	6 / 4 / 4	6 / 9 / 11
**V02**	6	87	88 / 84 / 78	87 / 84 / 89	3	2 / 2 / 4	4 / 7 / 7
**V03**	9	83	87 / 88 / 83	78 / 80 / 86	36	41 / 44 / 52	30 / 40 / 46
**V04**	12	96	98 / 98 / 96	94 / 98 / 96	56	56 / 58 / 61	55 / 69 / 64
**V05**	18	97	99 / 100 / 100	94 / 96 / 96	70	68 / 68 / 74	71 / 78 / 75
**V06**	24	97	99 / 100 / 100	94 / 98 / 96	78	74 / 74 / 78	83 / 89 / 89
**V07**	30	95	96 / 96 / 96	94 / 96 / 93	83	80 / 80 / 83	86 / 87 / 82
**V08**	36	92	96 / 98 / 100	87 / 87 / 86	81	83 / 86 / 87	80 / 82 / 79
**V09**	42	88	93 / 94 / 96	83 / 80 / 86	83	84 / 84 / 87	81 / 80 / 86
**V10**	48	86	94 / 96 / 100	77 / 80 / 79	82	89 / 92 / 96	74 / 80 / 79
**V11**	54	81	87 / 84 / 83	74 / 78 / 75	75	78 / 78 / 78	71 / 76 / 75
**V12**	60	79	89 / 92 / 100	67 / 71 / 61	75	84 / 90 / 96	65 / 71 / 61
**R12**	66	1	1 / 0 / 0	0 / 0 / 0	1	1 / 0 / 0	0 / 0 / 0
**V13**	72	71	76 / 80 / 91	65 / 64 / 61	70	74 / 78 / 91	64 / 62 / 57
**R13**	78	1	0 / 0 / 0	1 / 0 / 0	0	0 / 0 / 0	0 / 0 / 0
**V14**	84	62	65 / 66 / 70	59 / 56 / 50	62	63 / 66 / 70	59 / 56 / 50
**R14**	90	0	0 / 0 / 0	0 / 0 / 0	0	0 / 0 / 0	0 / 0 / 0
**V15**	96	48	51 / 48 / 57	45 / 47 / 39	47	49 / 46 / 57	45 / 47 / 39
**R15**	102	13	17 / 18 / 17	9 / 9 / 11	16	22 / 26 / 26	9 / 9 / 11
**V16**	108	46	48 / 56 / 65	43 / 38 / 36	44	45 / 54 / 65	43 / 38 / 36
**R16**	114	32	39 / 42 / 43	23 / 24 / 25	38	41 / 40 / 43	33 / 36 / 32
**V17**	120	54	59 / 56 / 65	48 / 49 / 36	52	56 / 54 / 65	46 / 47 / 32
**R17**	126	30	29 / 20 / 26	30 / 24 / 18	38	38 / 30 / 39	39 / 31 / 25
**V18**	132	378	41 / 34 / 35	33 / 31 / 21	37	39 / 32 / 35	35 / 33 / 25
**R18**	138	15	10 / 4 / 4	20 / 18 / 11	17	13 / 8 / 9	22 / 20 / 14
**V19**	144	11	11 / 8 7 4	10 / 9 / 4	9	9 / 6 / 0	10 / 9 / 4
**R19**	150	3	2 / 0 / 0	3 / 0 / 0	2	1 / 0 / 0	3 / 0 / 0
**V20**	156	0	0 / 0 / 0	0 / 0 / 0	0	0 / 0 / 0	0 / 0 / 0

Availability of the levodopa-induced dyskinesia information derived from the MDS-UPDRS-III and IV score at different assessment time points for the entire Parkinson's disease cohort as well as for the high and low resilience group. MDS-UPDRS-III = Movement Disorder Society - Unified-Parkinson's-Disease-Rating-Scale motor-score, IV = motor complications

Supplementary Table 9 - Characteristics of the dopamine transporter signal linear mixed model cohort

LMM DOPAMINE TRANSPORTER COHORT -4-YEAR FOLLOW-UP-			GROUP COMPARISON -HIGH VS. INTERMEDIATE VS. LOW RESILIENCE-			
	**Characteristics**	**Average +/- SD**	**High resilience**	**Intermediate resilience**	**Low resilience**	**p-value**
**DEMOGRAPHICS**	Number	115	41	42	32	
	Sex (m/w)	68/47	22/19	30/12	16/16	0.12
	Age	59.5± 4.0	59.1 ± 3.9	59.8 ± 3.9	59.5 ± 4.2	0.77

Patient characteristics of the entire linear mixed model cohort, for whom 4-years follow-up information of the dopamine transporter signal were available, as well as the group-specific information of patients with high, intermediate and low resilience. The last column indicates if there are significant between-group differences. LMM = Linear Mixed Model, SD = Standard Deviation
