# Supplementary material for: Structural underpinnings and long-term effects of resilience in Parkinson’s disease
Source: NPJ Parkinsons Dis. 2024 May 2;10:94. doi: 10.1038/s41531-024-00699-x (PMC11066097; doi:10.1038/s41531-024-00699-x)
Supplement: Supplementary file 2 — Reporting Summary [file 41531_2024_699_MOESM2_ESM.pdf]

nature portfolio

Corresponding author(s): Thilo van Eimeren
NPJPARKD-02045R1

Last updated by author(s): Mar 25, 2024

Reporting Summary

Nature Portfolio wishes to improve the reproducibility of the work that we publish. This form provides structure for consistency and transparency in reporting. For further information on Nature Portfolio policies, see our Editorial Policies and the Editorial Policy Checklist.

Statistics

For all statistical analyses, confirm that the following items are present in the figure legend, table legend, main text, or Methods section.

n/a	Confirmed	
☐	☒	The exact sample size (*n*) for each experimental group/condition, given as a discrete number and unit of measurement
☐	☒	A statement on whether measurements were taken from distinct samples or whether the same sample was measured repeatedly
☐	☒	The statistical test(s) used AND whether they are one- or two-sided<br>*Only common tests should be described solely by name; describe more complex techniques in the Methods section.*
☐	☒	A description of all covariates tested
☐	☒	A description of any assumptions or corrections, such as tests of normality and adjustment for multiple comparisons
☐	☒	A full description of the statistical parameters including central tendency (e.g. means) or other basic estimates (e.g. regression coefficient) AND variation (e.g. standard deviation) or associated estimates of uncertainty (e.g. confidence intervals)
☐	☒	For null hypothesis testing, the test statistic (e.g. *F*, *t*, *r*) with confidence intervals, effect sizes, degrees of freedom and *P* value noted<br>*Give P values as exact values whenever suitable.*
☒	☐	For Bayesian analysis, information on the choice of priors and Markov chain Monte Carlo settings
☐	☒	For hierarchical and complex designs, identification of the appropriate level for tests and full reporting of outcomes
☐	☒	Estimates of effect sizes (e.g. Cohen's *d*, Pearson's *r*), indicating how they were calculated

Our web collection on statistics for biologists contains articles on many of the points above.

Software and code

Policy information about availability of computer code

Data collection	NA
Data analysis	NA

For manuscripts utilizing custom algorithms or software that are central to the research but not yet described in published literature, software must be made available to editors and reviewers. We strongly encourage code deposition in a community repository (e.g. GitHub). See the Nature Portfolio guidelines for submitting code & software for further information.

Data

Policy information about availability of data

All manuscripts must include a data availability statement. This statement should provide the following information, where applicable:

- Accession codes, unique identifiers, or web links for publicly available datasets
- A description of any restrictions on data availability
- For clinical datasets or third party data, please ensure that the statement adheres to our policy

The data used for this study are publicly available via the PPMI website. Unique identifiers of the subjects included in this study can be found in Supplementary Table 4.

Research involving human participants, their data, or biological material

Policy information about studies with human participants or human data. See also policy information about sex, gender (identity/presentation), and sexual orientation and race, ethnicity and racism.

Reporting on sex and gender	We used the term sex throughout the manuscript to refer to the participants' biological attributes. Analyses included sex as a covariate to account for biological differences between male and female participants. Sex information was acquired via PPMI (for further information, see https://www.ppmi-info.org/). Our study included 89 male and 62 female participants. Sex-based differences were beyond the scope of our study, which targeted gender-unspecific resilience mechanisms in Parrkinson's disease patients.
Reporting on race, ethnicity, or other socially relevant groupings	We did not group study participants in regard to ethnicity or other socially relevant aspects.
Population characteristics	Our study considered age and sex as essential covariables for all analyses. Further, total intracranial volume and study site were regarded as relevant for structural MRI data analysis. Lastly, longitudinal Levodopa-daily equivalent dose and baseline Montreal Cognitive Assessment Scores were included in models investigating resilience-dependent decline rates in motor functioning in Parkinson's disease patients.
Recruitment	https://www.ppmi-info.org/
Ethics oversight	Ethical approval and written informed consent according to the Declaration of Helsinki for all patients was obtained from the respective PPMI sites.

Note that full information on the approval of the study protocol must also be provided in the manuscript.

Field-specific reporting

Please select the one below that is the best fit for your research. If you are not sure, read the appropriate sections before making your selection.

☒ Life sciences ☐ Behavioural & social sciences ☐ Ecological, evolutionary & environmental sciences

For a reference copy of the document with all sections, see nature.com/documents/nr-reporting-summary-flat.pdf

Life sciences study design

All studies must disclose on these points even when the disclosure is negative.

Sample size	We included all patients from the Parkinson's Progression Marker Initiative that fit our inclusion criteria. This led to a cohort size of 151 patients and subgroups of at least 45 patients. Only in the longitudinal analysis investigating the resilience level-dependent decline rates in motor function over a seven-year follow-up period, group sizes were below n=45, ranging between n=18 and n=27. Considering the central limit theorem, the sample distribution should converge to a normal distribution at a sample size of 30. Therefore, sample sizes above 30 are adequate to infer population behaviour based on the sample results. For the longitudinal analysis, we decided to only include patients with complete follow-up periods to allow an accurate model fit over the whole time interval. This led to smaller group sizes. However, it must be considered that n=18 participants correspond to 144 data points.
Data exclusions	We excluded all patients younger than 50 and older than 66.5 years to minimize genetic and age-related influences on the results. Further, patients needed baseline imaging (DaT SPECT, T1 MRI) and clinical data (UPDRS-III assessment at baseline and at least one PASE score). For longitudinal analysis, patients required a four-year follow-up period for DaT SPECTS and a seven-year follow-up for UPDRS-III assessments. Regarding the structural network analysis, only patients allocated to the high or low resilience group were used for network comparison.
Replication	For the network analysis, we performed validation in the form of a leave-one-out cross-validation to ensure the robustness of the results.
Randomization	In this study, no experimental groups were defined. Therefore, randomization was not applicable.
Blinding	Since we downloaded the data from an online database (PPMI), blinding was unnecessary in the data acquisition process. Further, the grouping of the patients was based on a predefined model and, therefore, was independent of the experimenter performing the analysis.

Reporting for specific materials, systems and methods

We require information from authors about some types of materials, experimental systems and methods used in many studies. Here, indicate whether each material, system or method listed is relevant to your study. If you are not sure if a list item applies to your research, read the appropriate section before selecting a response.

Materials & experimental systems

n/a	Involved in the study
☒	☐ Antibodies
☒	☐ Eukaryotic cell lines
☒	☐ Palaeontology and archaeology
☒	☐ Animals and other organisms
☐	☒ Clinical data
☒	☐ Dual use research of concern
☒	☐ Plants

Methods

n/a	Involved in the study
☒	☐ ChIP-seq
☒	☐ Flow cytometry
☐	☒ MRI-based neuroimaging

Clinical data

Policy information about clinical studies

All manuscripts should comply with the ICMJE guidelines for publication of clinical research and a completed CONSORT checklist must be included with all submissions.

Clinical trial registration: NCT01141023

Study protocol: https://www.ppmi-info.org/study-design/research-documents-and-sops

Data collection: https://www.ppmi-info.org/

Outcomes: https://www.ppmi-info.org/

Plants

Seed stocks: *Report on the source of all seed stocks or other plant material used. If applicable, state the seed stock centre and catalogue number. If plant specimens were collected from the field, describe the collection location, date and sampling procedures.*

Novel plant genotypes: *Describe the methods by which all novel plant genotypes were produced. This includes those generated by transgenic approaches, gene editing, chemical/radiation-based mutagenesis and hybridization. For transgenic lines, describe the transformation method, the number of independent lines analyzed and the generation upon which experiments were performed. For gene-edited lines, describe the editor used, the endogenous sequence targeted for editing, the targeting guide RNA sequence (if applicable) and how the editor was applied.*

Authentication: *Describe any authentication procedures for each seed stock used or novel genotype generated. Describe any experiments used to assess the effect of a mutation and, where applicable, how potential secondary effects (e.g. second site T-DNA insertions, mosiacism, off-target gene editing) were examined.*

Magnetic resonance imaging

Experimental design

Design type: Structural T1-weighted MRI

Design specifications: Not applicable given structural imaging.

Behavioral performance measures: Not applicable given structural imaging.

Acquisition

Imaging type(s): Structural T1-weighted MRI

Field strength: 3T

Sequence & imaging parameters: https://www.ppmi-info.org/sites/default/files/docs/PPMI2.0_002_MRI_TOM_Final_v3.0_20210727.pdf

Area of acquisition: Whole brain

Diffusion MRI: ☐ Used ☒ Not used

Preprocessing

Preprocessing software: SPM12 (Preprocessing)
CAT12.7 (Segmentation)

Normalization	Normalized to MNI space.
Normalization template	Data were normalized to the Montreal Neurological Institute space.
Noise and artifact removal	Assessment of image quality was performed in CAT12 using the standard pipeline.
Volume censoring	SPM Version 12

Statistical modeling & inference

Model type and settings	ANCOVA
Effect(s) tested	High vs. low resilience

Specify type of analysis: ☒ Whole brain ☐ ROI-based ☐ Both

Statistic type for inference (See Eklund et al. 2016)	Voxel-wise gray-matter volume (parameters see below)
Correction	All clusters significant at cluster-level after FWE-correction (p<0.05) with an initial p-value set at p<.001 were reported.

Models & analysis

n/a	Involved in the study
☒	☐ Functional and/or effective connectivity
☐	☒ Graph analysis
☒	☐ Multivariate modeling or predictive analysis

Graph analysis	Structural covariance measures were used to gain binarized graphs. Based on group levels, we compared regional measures of betweenness centrality hubs.
